# Molecular Basis of Siglec‑7 Recognition by Neisseria meningitidis Serogroup Y CPS: Implications for Immune Evasion

**DOI:** 10.1021/jacsau.5c00214

**Published:** 2025-04-30

**Authors:** Cristina Di Carluccio, Tania Gerpe Amor, Maria Pia Lenza, Alessandro Antonio Masi, Celeste Abreu, Viviana Longo, Francesco Albano, Ferran Nieto-Fabregat, Paola Salvatore, Geppino Falco, Darielys Santana-Medero, Marco Fragai, Yvette van Kooyk, Antonio Molinaro, Yury Valdes-Balbin, Ondřej Vaněk, Vicente Verez-Bencomo, Roberta Marchetti, Fabrizio Chiodo, Alba Silipo

**Affiliations:** † Department of Chemical Sciences, 9307 University of Naples Federico II, via Cinthia 4, 80126 Naples, Italy; ‡ Department of Biochemistry, Faculty of Science, 112302 Charles University, Hlavova 2030/8, 12800 Prague, Czech Republic; § Stem Cell Biology Laboratory, Department of Biology, University of Naples Federico II, 80131 Naples, Italy; ∥ Department of Molecular Medicine and Medical Biotechnology, University of Naples Federico II, via Pansini, 5, 80131 Naples, Italy; ⊥ 329611 Finlay Vaccine Institute, 21st Ave. N◦ 19810 between 198 and 200 St, Atabey, Playa, 10400 Havana, Cuba; # Magnetic Resonance Centre (CERM), CIRMMP and Department of Chemistry “Ugo Schiff”, 9300 University of Florence, 50019 Sesto Fiorentino, Italy; ∇ Department of Molecular Cell Biology and Immunology, Amsterdam UMC, Vrije Universiteit Amsterdam, Amsterdam 1081 HV, The Netherlands; ○ Institute of Biomolecular Chemistry, National Research Council (CNR), via Campi Flegrei, 34, 80078 Pozzuoli, Naples, Italy

**Keywords:** Neisseria meningitidis serogroup Y CPS, Siglec-7, immune evasion, binding studies, sialic acid

## Abstract

Siglecs, sialic-acid-binding immunoglobulin-like lectins, are key immune cell receptors that recognize sialic acid residues on cell surfaces. Pathogens and tumor cells exploit Siglecs to evade immune responses and modulate immunity, contributing significantly to infectious disease and cancer pathogenesis. Siglec-7, primarily expressed on natural killer (NK) cells, functions as an inhibitory receptor, tightly regulating the immune activity. This study investigates the interaction between Siglec-7 and the capsular polysaccharide (CPS) of Neisseria meningitidis serogroup Y (Men-Y), a bacterium whose sialylated CPS is critical for virulence. We demonstrate that Men-Y CPS binds to inhibitory Siglec-7, potentially dampening immune recognition. We employed a multifaceted approach, combining biochemical and biophysical techniques to dissect this interaction. Enzyme-linked immunosorbent assays (ELISAs) and fluorescence titrations quantified the binding specificity and affinity. Ligand- and protein-based nuclear magnetic resonance (NMR) spectroscopy, coupled with computational modeling, provides detailed molecular insights. We highlight the critical influence of the Men-Y CPS conformation and sialic acid presentation on Siglec-7 binding. The specific arrangement of α-2,6-linked sialic acids on the CPS is crucial for Siglec-7 binding, demonstrating the importance of the CPS 3D structure. Preliminary immunological assays using stimulated U937 cells (a promonocytic cell line) further support the immunomodulatory role of Siglec-7 mediated by Men-Y CPS. These results offer valuable insights into the development of targeted therapeutic strategies against bacterial infections.

## Graphical abstract

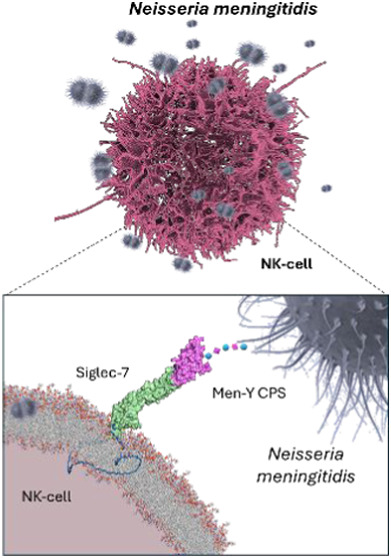

## Introduction

Siglecs (sialic-acid-binding immunoglobulin-type lectins) represent an important family of immunomodulatory receptors present primarily on immune cells. The human genome encodes multiple Siglec members, each possessing distinct ligand-binding specificities and intracellular signaling motifs; while certain Siglecs trigger immune responses upon ligand binding, most act as inhibitory receptors, tempering immune activation. This sophisticated system highlights their intricate role in modulating immune responses and underscores their significance in both health and disease. Siglecs are distinguished by their unique ability to bind specifically to sialic acid residues (Sias) found in glycoproteins and glycolipids. Sialic acids, a group of nine-carbon sugars, typically decorate the ends of glycan chains on cell surfaces and secreted proteins, playing pivotal roles in cellular functions like intercellular communication, signaling, and immune recognition.^1^ Interestingly, this crucial feature can be exploited by tumor cells and pathogens, including bacteria and viruses, to evade immune detection and clearance by capitalizing on host cell machinery, particularly targeting sialic acid residues. Indeed, these pathogens can mimic host cell glycans by expressing sialylated molecules on their surfaces, thereby deceiving Siglecs to attenuate host immune responses and facilitate dissemination.^2^ Pathogens such as Escherichia coli K1, Neisseria meningitidis, and Neisseria gonorrheae, group B *Streptococcus* (GBS), and Campylobacter jejuni, and viruses such as HIV-1, SARS-CoV-2, and Ebola engage Siglecs by sialylated envelope components. In bacteria, this mimicry is mediated by envelope glycoconjugates such as sialylated capsules and/or lipopolysaccharide (LPS), or, in some cases, by modified flagellin.^3^

Siglec-7, a member of human CD33-related inhibitory Siglec receptors, acts as a negative regulator of the innate immune system.^4^ Siglec-7 is mainly expressed on natural killer (NK) cells and it consists of an extracellular N-terminal V-set sialic-acid-binding domain, followed by two C2-type Ig-like domains that outdistance the carbohydrate binding region from the cellular surface and an immunoreceptor tyrosine-based inhibition motif (ITIM)^5^ in the cytosolic region. Siglec-7 preferentially binds α-(2,8)-linked disialylated ligands, generally found as terminal portions of gangliosides, such as GD3, and internally branched α-(2,6)-sialyl residues present in DSGb5 and DSLc4 gangliosides.^6^ The involvement of Siglec-7 in various diseases, including cancer, infection, and autoimmune disorders, combined with its immunomodulatory capacity, positions it as a promising therapeutic target and a novel glycoimmune checkpoint.^7,8^ Siglec-7 is engaged by Candida albicans, GBS, C. jejuni and Pseudomonas aeruginosa, among others, to evade immune surveillance, promoting successful bacterial colonization and critical tolerance to the pathogen.^3,9^

Neisseria meningitidis (*Nm*) is a Gram-negative human pathogen and etiological agent of meningococcal meningitis, a potentially fatal infection affecting the membranes surrounding the brain and spinal cord. It can cause large-scale epidemics.^10^ The capsular polysaccharides (CPS) surrounding the *Nm* cell envelope represent major virulence factors, playing a crucial role in *Nm* pathogenicity.^11^ As such, they are the main component of Meningococcal vaccines, either as pure CPS or as protein-conjugate vaccines.^12^ *Nm* holds 13 serogroups,^13^ categorized on their CPSs into three pairs: Men-A and Men-X, whose CPSs are composed of phosphodiester polymers of amino sugars; Men-B and Men-C, whose CPSs consist of α-2,8 and α-2,9 sialic acid homopolymers, respectively; Men-Y and Men-W, whose CPSs contain sialic acid as [4)-α-Neu5Ac-(2→6)-α-Glc-(1→]*_n_* and [4)-α-Neu5Ac-(2→6)-α-Gal-(1→]*_n_*, respectively (Table S1).^14^ Recently, the USA Centers for Disease Control and Prevention issued a health advisory to alert for an increase in invasive meningococcal cases, mainly due to Men-Y. In addition, the neuronal damage caused by bacterial meningitis has also been correlated to the onset of neurodegenerative diseases, while the study of the molecular interactions and pathways linking infections with neuronal damage remains mostly unexplored.

Most of the studies describing carbohydrates-based host–Meningococcal interactions deal with the bacterial LPS,^15,16^ and recently the *Neisseria* adhesin A (NadA)—a meningococcal surface protein included in vaccines—has been reported to bind Siglec-5 and Siglec-14.^17^ The widespread presence of Sias within *Nm* CPS (Table S1) suggests a potential mechanism for immune evasion, whereby these bacteria target host immune receptors such as Siglecs to trigger inhibitory signaling.^18^ To gain deeper insights into the immunomodulatory roles of *Nm* CPS from both host–pathogen interaction and vaccinology perspectives, we focused on *Nm* serogroup Y and demonstrated its recognition by Siglec-7. This finding highlights the ability of immunomodulatory inhibitory receptors to be exploited by bacterial envelope glycans, thereby facilitating immune evasion. Building upon promising preliminary results from ELISA solid-phase assays, which demonstrated the ability of Men-Y CPS to bind exclusively to inhibitory Siglec-7 and no other Siglecs or human C-type lectins, we delved deeper into this interaction at both the molecular and immunological levels. A combination of NMR, computational, and biophysical techniques was employed to evaluate the factors governing the selective recognition of Siglec-7 to Men-Y CPS and to characterize the binding interface, thereby elucidating the molecular basis of this interaction. Our outcomes hold promise not only for understanding the pathogenicity of *Nm* but also for advancing our knowledge at the molecular level of bacterial immune evasion mechanism.^19,20^

## Methods

### Protein Expression and Purification

The full extracellular domain of Siglec-7 (Gly18-Gly354) with a C-terminal histidine tag was expressed in suspension-adapted human HEK293S GnTI^–^ cells, as previously indicated.^21,22^ Siglec-7 FED was produced as a glycoprotein containing oligomannose-type glycans to avoid interferences from sialic acids during the binding with the ligands. ExCELL 293 serum-free cell culture medium (Merck) was used for cell line cultivation and transfection. The protein was purified from the cell culture supernatant by immobilized metal affinity chromatography (IMAC) followed by size-exclusion chromatography (SEC). The carbohydrate recognition domain of Siglec-7 (Gly18-His148) was expressed in LB and M9 culture medium. The inclusion bodies were resuspended in 8 M urea lysis buffer; the soluble protein was then subjected to IMAC purification on a HisTrap FF. The protein was refolded and purified by SEC on a HiLoad 26/60 Superdex 75 pg (GE Healthcare) coupled to an AKTA Go FPLC system. Expression and purification of Siglec-7 CRD in E. coli was performed as previously reported.^23^

### Partial CPS Depolymerization

CPS was depolymerized in acetate buffer (pH of 4,5) for 8 h at 90°, and the solution was then neutralized with NaOH 1 M and lyophilized. The depolymerized sample was then separated on a size-exclusion chromatography column TSK-40.

### Ligand-Based NMR Experiments

Nuclear magnetic resonance (NMR) spectra were acquired on a Bruker 600 MHz Avance Neo instrument equipped with a cryo-probe. NMR samples were dissolved in deuterated PBS buffer; [D4] (trimethylsilyl)­propionic acid, sodium salt (TSP, 10 μM) was used as internal reference to calibrate all of the spectra. Data acquisition and processing were analyzed by using TOPSPIN 4.4 software. Homonuclear 2D ^1^H–^1^H NOESY experiments were carried out by using data sets of 4096 × 900 points and mixing times of 100–300 ms. 2D homonuclear spectra were recorded with data sets of 4096 × 900 (t1 × t2) points and the data matrix processed with zero-filling in the F1 dimension up to 4096 × 2048 points. To improve the resolution, a cosine-bell function was used before the Fourier transformation in both dimensions. Heteronuclear single quantum coherence (HSQC) experiments were carried out with setting data points of 2048 × 600. A protein:ligand ratio of 1:40–100 and a saturation time of 2 s were used with the on-resonance pulse at 7.5 ppm and the off-resonance pulse at 40 ppm. By using these conditions, no STD signals were observed in the control STD NMR spectrum of the ligand alone. A train of 50 ms (field strength of 21 Hz) Gaussian-shaped pulse with an attenuation of 60 db has been used to saturate the protein. The epitope mapping of the partially depolymerized CPS Men-Y was achieved by the calculation of the ratio (*I*_0_ – *I*_sat_)/I_0_, where (*I*_0_ – *I*_sat_) is the intensity of the signal in the STD NMR spectrum and *I*_0_ is the peak intensity referred to the unsaturated reference spectrum (*off*-resonance). For overlapped protons, the combination with computational analysis helped to define the epitope mapping.

### NMR Protein Assignment

Triple resonance experiments for protein NMR assignment HNCA, HNCACB, HNCO, and CBCAcoNH were recorded at 298 K on a Bruker’s Avance NEO 900 MHz spectrometer, equipped with a TCI cryo-probe. 3D HNcaCO was recorded at 298 K on a Bruker’s Avance 500 MHz spectrometer. 93% of the amino acid sequence from Y26 to T147 was assigned, excluding the 5 proline residues.^23^ Data acquisition and processing was performed on TOPSPIN 4.1.1 software, and spectra were analyzed by using CARA (Computer Aided Resonance Assignment) software.

### Protein-Based NMR Experiments

For ligand-binding studies, 2D ^1^H–^15^N HSQC NMR experiments were recorded on samples of 200 μM [U–^15^N] Siglec-7 CRD in 200 μL of aqueous buffered solution (20 mM potassium phosphate, pH 7.4, 50 mM NaCl, 0.01% NaN_3_, 1 mM protease inhibitors, and 10% D_2_O in a 3 mm NMR tube). Experiments were acquired on a Bruker’s Avance NEO 600 MHz spectrometer, equipped with a triple resonance cryo-probe. The spectra were acquired using 32 scans with 2048 data point in t2 and 128 increments in the indirect dimension (t1), a recycle delay of 2 s, and the temperature was kept at 298 K. The interaction was investigated by titrating 100 μM Siglec-7 CRD with increasing amounts of ligands to reach large excess with respect to protein (1:20). 2D ^1^H–^15^N HSQC spectra were added upon the addition of each ligand aliquot. Data acquisition and processing were performed with TOPSPIN 4.1.1 software and the spectra were analyzed using CARA. Chemical Shift Perturbations (CSP) were evaluated with the formula: Δδ = 1/2√(⟨Δδ⟩_(H^∧^2) + ⟨(⟨Δδ⟩_N/5)⟩^∧^2). The threshold of CSPs was set based on the second standard deviation of the CSP values measured at 1:10 protein/ligand molar ratio (with a protein concentration of 100 μM). The threshold of intensity decreases was evaluated as the average value minus the first standard deviation of the values of the last titration point (1:20 ratio).

### ELISA: C-type lectins ELISA

A solution of 50 μL of Men-Y and Men-W CPS from N. meningitidis isolates (Neu5Ac-Glc) and Neu5Ac-Gal 50 μg/mL in PBS (10 mM, pH = 7.4) provided by Finlay Institute Cuba was used to coat the Nunc MaxiSorp plate 2 h at room temperature. After being discarded and washed (2 × 150 μL) with calcium and magnesium-containing buffer TSM [20 mM tris­(hydroxymethyl)­aminomethane (Tris)-HCl, pH 8.0; 150 mM NaCl; 1 mM CaCl_2_; 2 mM MgCl_2_], the wells were blocked with 100 μL of 1% BSA (Sigma-Aldrich, lyophilized powder, ≥96%, agarose gel electrophoresis) in TSM at 37 °C for 30 min. The blocking solution was discarded and 50 μL of different C-type-lectins human-Fc at 1 μg/mL in an assay buffer (TSM, 0.5% BSA) were added to the wells. After 1 h at room temperature, the wells were washed with TSM (2 × 150 μL) and then 100 μL of antihuman horseradish peroxidase (0.3 μg/mL, Goat anti-Human IgG-HRP from JacksonImmuno) was added. After 30 min at room temperature, the wells were washed with TSM (2 × 150 μL). Finally, 100 μL of substrate solution (3,3′,5,5′-tetramethylbenzidine, TMB, in citric/acetate buffer, pH = 4, and H_2_O_2_) was added and after max 15 min incubation at room temperature, the reaction was stopped with 50 μL of H_2_SO_4_ (0.8 M) and the optical density (OD) was measured at 450 nm in an ELISA reader. Polyacrylamide polymers (PAA), functionalized with different glycans, were purchased from Lectinity, MW approximately 20 kDa, carbohydrate content around 20% mol. Galβ1-4­(Fucα1-3)­GlcNAcβ-OCH2CH2CH2NH2 (PAA-LeX, positive control for DC-SIGN) and GalNAcα-OCH2CH2CH2NH2 (PAA-Tn, positive control for MGL), we used a concentration of 40 μg/mL PAA-glycoconjugates to coat the ELISA wells. The experiment was performed three times in duplicate with similar results. Data were normalized over the binding signal from the positive controls used for each C-type lectin (set as 100% binding). ***Siglecs-ELISA***: A solution of 50 μL of Men-Y and Men-W CPS from N. meningitidis isolates (Neu5Ac-Glc) and Neu5Ac-Gal 50 μg/mL in PBS (10 mM, pH = 7.4) provided by Finlay Institute Cuba was used to coat the Nunc MaxiSorp plate 2 h at room temperature. After discarding and washing (2 × 150 μL) with Hanks’ Balanced Salt solution (Gibco HBSS), the wells were blocked with 200 μL of carbo-free blocking solution (Vector Laboratories, Catalog No. NC9977573) at 37 °C for 30 min. The blocking solution was discarded and 50 μL of different human Siglecs-Fc constructs (recombinant human chimera purchased from R&D Systems Netherlands) at 5 μg/mL (for Siglec-9 100 ng/mL) in an assay buffer (carbo-free solution) were added to the wells. After 2 h at room temperature, under gentle shaking (150 rot/min), the wells were washed with HBSS (2 × 150 μL) and then 100 μL of antihuman horseradish peroxidase (0.3 μg/mL, Goat anti-Human IgG-HRP from JacksonImmuno) was added. After 30 min at room temperature, the wells were washed with HBSS (2 × 150 μL). Finally, 100 μL of substrate solution (3,3′,5,5′-tetramethylbenzidine, TMB, in citric/acetate buffer, pH = 4, and H_2_O_2_) was added and after max 15 min incubation at room temperature, the reaction was stopped with 50 μL of H_2_SO_4_ (0.8 M) and the optical density (OD) was measured at 450 nm in an ELISA reader. Polyacrylamide polymers (PAA) functionalized with different glycans were purchased from Lectinity, MW approximately 20 kDa, carbohydrate content around 20% mol. Neu5Acα6′Lac-C2-PAA 0063a-PA (PAA-Sia 2,6), Neu5Acα3′Lac-Gly-PAA (PAA-Sia 2,3), we used a concentration of 40 μg/mL PAA-glycoconjugates to coat the ELISA wells. The experiment has been performed three times in duplicate with similar results. Data were normalized over the binding signal from the positive controls used for each Siglec (set as 100% binding).

### Fluorescence Analysis

Stationary-state fluorescence spectra were collected on a Fluoromax-4 spectrophotometer (Horiba, Edison, NJ, USA). Emission spectra were recorded in the range of 310–450 nm with excitation at 295 nm. The slit widths were set to 5 nm for excitation and to 5 nm for the emission wavelength. A quartz cuvette with a path length of 1 cm and a volume of 0.2 mL was used. The starting solution of Siglec-7 CRD protein, prepared at a fixed concentration of 4 μM in PBS buffer (pH 7.4), was titrated by adding small aliquots of a stock solution of 100 μM of the ligands Men-Y and Men-W capsular polysaccharides from N. meningitidis, already depolymerized and purified. The experiments were carried out at 25 °C. For all ligands analyzed, the fluorescence intensity was quenched. The binding curve was obtained by fitting the data using nonlinear regression with a 1:1 binding model using the function described by Ribeiro et al.^24^ (plotting the ratio between the fluorescence intensity at each addition of ligand (*F*) and the fluorescence intensity of the protein in the absence of ligand (*F*_0_) against the total ligand concentration [*L*] in μM).

### *In Silico* Methods

#### Ligand Preparation for Computational Analysis

3D coordinates for multiple repeating units of Men-Y were generated by using the GLYCAM database. Subsequently, the ligand geometries underwent optimization through MD simulations. *Docking calculations.* Docking calculations of different-length oligosaccharides representing the Men-Y repeating unit structure were performed using AutoDock 4.2.^25^ The analysis of the docking poses was performed with AutoDock 4.2 Tools. The docking protocol was validated by carrying out the docking of the crystal structure from the Siglec-7 2HRL pdb ID. A total of 200 runs using the Lamarckian Genetic algorithm was performed, with a population size of 100. After docking, the 100 solutions were clustered in groups with a root-mean-square deviation less than 1.0 Å. The clusters were ranked as the lowest energy representative of each one. *MD simulation.* Molecular dynamics calculations were performed within the AMBER 18 ^26^ software package using explicit water with the following force fields: Glycam06j-1 ^27^ for the glycans, FF14SB^28^ for the protein, and Gaff^29^ for organic moieties. For the protein preparation, missing hydrogen atoms were added, protonation state of ionizable groups were added (Na^+^ and Cl^–^), and cap termini were computed by using Pymol.^30^ The input files were generated using the tleap modules of the AMBER packages, the minimization step was performed by using Sander module, and the productions of the Molecular Dynamic calculations were performed by using the PMEMD module. The corresponding molecules (free and bound-state different serogroup Y R.U sizes) were position within a truncated octahedral box of TIP3P water of proper size and the remote interactions were calculated using a cut off of 15 Å, and counterions (Na^+^ and Cl^–^) were added to neutralize the whole system. After the preparation of the input files, an energy minimization process was performed to refine the initial structure. The calculations employ SHAKE for the C–H bond and 1 fs of integration step. Periodic boundary conditions were applied as well as the smooth particle mesh Ewald method to represent the electrostatic interactions, with a grid space of 1 Å. The system was minimized, first, by holding the solute fixed, while a second minimization performs the entire system. Afterward, the whole system was slowly heated using constant pressure and constant volume from 0 to 300 K using a weak restraint on the solute, and then, the system was equilibrated at 300 K using a constant pressure and removing the weak restraints that were put in the solute. The system coordinates were saved and used for 100 ns simulation using the PMEMD module implemented in AMBER. Coordinate trajectories were recorded each 2 ps throughout production runs, yielding and assembling 10,000 structures for each complex, which were later analyzed. The stability of energy, pressure, temperature, and other thermodynamic parameters were monitored along the trajectory, and then, RMSD torsion values, cluster distances, and hydrogen bonds were extracted by using the Cpptraj module. This module was useful for the analysis process of the trajectories and coordinate files that were created during the MD simulation. VMD^31^ software and Pymol software were used to visualize the trajectory.

### Immunological Assays

U937 (ATCC CRL-1593.2TM; Manasas, VA, USA) and THP1 monocytes (ATCC TIB-202; Manasas, VA, USA) were cultured in RPMI-1640 with l-glutamine, supplemented with 10% inactivated fetal bovine serum (FBS; Gibco, Carlsbad, CA, USA), 100 U/mL penicillin and 100 g/mL streptomycin (Gibco, Carlsbad, CA, USA), 1X Normocin (Invivogen, San Diego, CA, USA), and 0.05 mM 2-mercaptoethanol (Gibco, Carlsbad, CA, USA) in a 5%-CO_2_ humidified incubator at 37 °C. To induce cell attachment and naïve macrophage differentiation, we applied a specific protocol for U937 cells and THP-1 cells. Then, 2 × 10^5^ U937 cells were seeded in a 12-well plate and treated with phorbol 12-myristate 13-acetate (PMA) at 100 ng/mL concentration for 48 h, followed by a recovery phase of additional 48 h. At the same time, 2 × 10^5^ THP-1 cells were treated with 20 nM PMA for 24 h, followed by 72 h recovery phase. Subsequently, both U937-PMA cells and THP1-PMA cells were incubated with varying concentrations (0.1 μg/mL and 1 μg/mL) of capsular polysaccharides (CPS) derived from Mer-Y or lipopolysaccharide (LPS) and then maintained at 37 °C for 16 h. After incubation, the cells were washed with PBS before being directly lysed on a plate using Norgen Biotek RNA extraction kit (Thorold, Ontario, Canada), following the manufacturer’s protocol. The extracted RNA (1 μg) was reverse-transcribed using the Quantitect Reverser transcription Kit (Qiagen, Hilden, Germany). For real-time quantitative PCR (qPCR), 10 ng of cDNA was utilized in a 10 μL PowerUP SYBR Green Master mix PCR kit (Thermo Fisher, Waltham, Massachusetts, USA) reaction, employing primers here listed and analyzed using the AriaMX system (Agilent, Santa Clara, California, USA). Gene expression was determined by the ΔΔCt method, normalizing TNFα and IL-8 gene expression to β-actin, before comparing to the untreated control (10.3390/ijms22179379).

Human IL-8: FW—ATGACTTCCAAGCTGGCCGT—RV—TACAATAATTTCTGTGTTGGC

Human TNFα: FW—GTTGTAGCAAACCCTCAAGCTG—RV—GAGGTACAGGCCCTCTGAT

Human β-actin: FW—CCGACAGGATGCAGAAGGAG—RV—GCCTAGAAGCATTTGCGGTG

## Results

The strategic approach to evaluate the interaction of Siglec-7 with Men-Y CPS was to combine NMR spectroscopy with other biophysical and computational methods. To this aim, the CPS was isolated, purified and the structure was confirmed by NMR (Figure 1 and Table S1), revealing the following disaccharide repeating unit made up of glucose and sialic acid [4)-α-Neu5Ac-(2→6)-α-Glc-(1→]*_n_* and a minor acetylation pattern on O9 of sialic acid (Sia) (below 20%, see the Supporting Information).^32,33^ Exploiting the lability of the Sias glycosidic linkage, partial depolymerization was performed, yielding oligosaccharides with different numbers of repeating units. These oligosaccharides were subsequently purified and subjected to binding studies (Figure S1). Siglec-7 CRD (carbohydrate recognition domain) was produced in E. coli as [U-^15^N-^13^C] Siglec-7 and the amino acid resonances, previously assigned to provide the fingerprint of the protein, were used to perform protein-based NMR binding studies.^23^ Conversely, the full extracellular domain of Siglec-7 produced in HEK293S cells was instead used for ligand-based NMR binding experiments.

**Figure 1:**
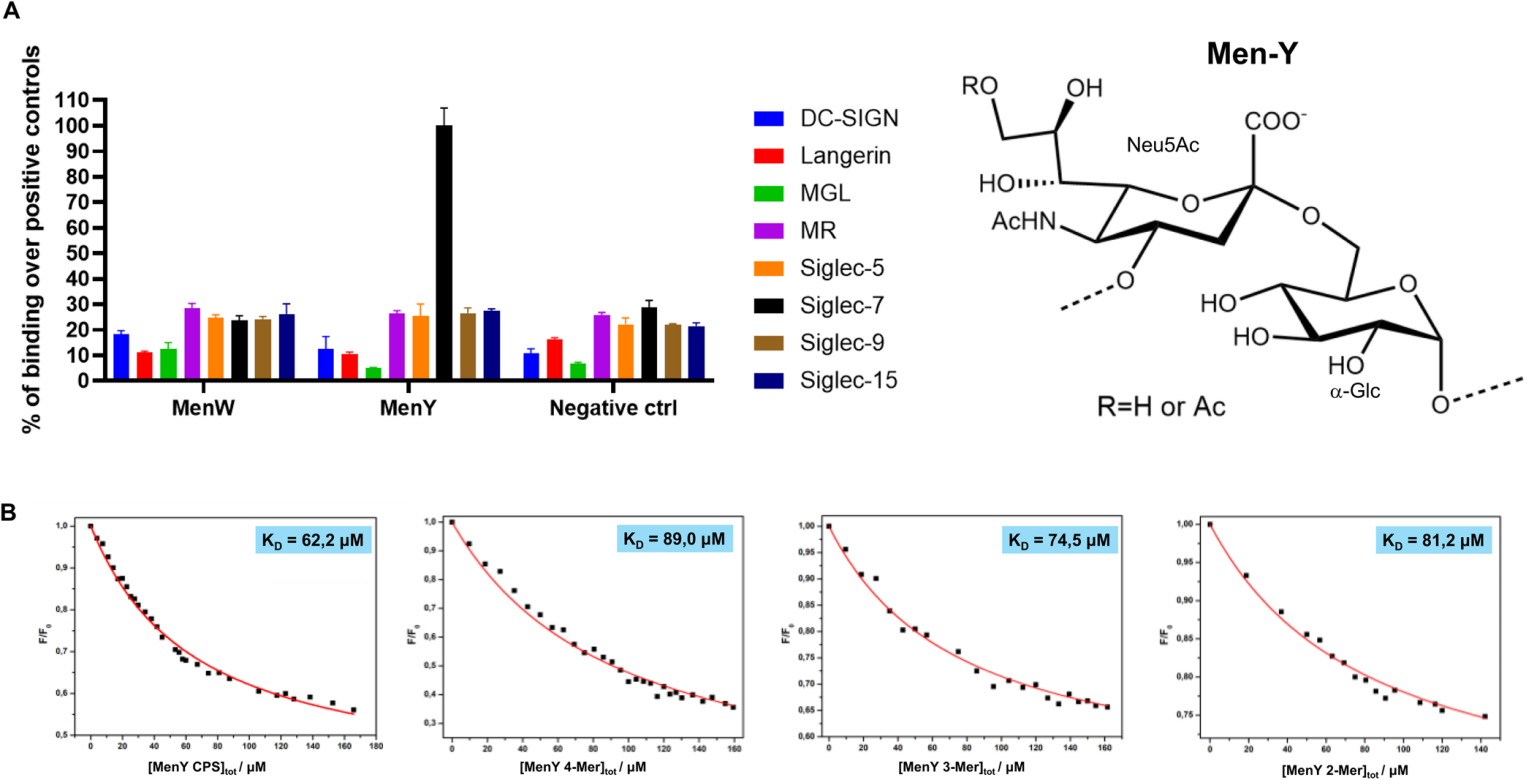
(A) ELISA solid-phase assay performed between different C-type lectins and Siglecs-Fc. Men-Y CPS showed a strong and selective binding to Siglec-7 compared to Men-W. Polyacrylamide polymers (PAA) coated with different glycans were used as positive controls. Wells were coated with the reported CPS and the binding to different C-type lectins and Siglecs was evaluated. The experiment has been performed three times in duplicate with similar results. Data are normalized over the binding signal from the positive controls used for each lectin (set as 100% binding). Error bars indicate standard deviations. OD: Optical density. (B) Fluorescence titration of Siglec-7 upon the addition of increasing concentrations of Men-Y CPS and oligos (4-Mer, 3-Mer, and 2-Mer). The emission spectra were recorded by using an excitation wavelength of 295 nm and a temperature of 25 °C.

### ELISA and Fluorescence Experiments

The profile of Men-Y CPS binding by Siglec-7 was initially detected by a solid-phase enzyme immunoassay (ELISA solid-phase assay), and subsequently by steady-state fluorescence analysis to determine the binding affinities. In the ELISA assay, different recombinant soluble forms of Siglecs (Siglec-5, 7, 9, and 15) and human C-type lectins (DC-SIGN, Langerin, MGL and MR) were used with their entire extracellular region fused to the Fc region of human IgG1. As depicted in Figure 1A, the ability of Siglec-7 to recognize Men-Y and not Men-W ([4)-α-Neu5Ac-(2→6)-α-Glc-(1→]*_n_* and [4)-α-Neu5Ac-(2→6)-α-Gal-(1→]*_n_*, respectively) was demonstrated in enzyme immunoassay experiments, indicating that Siglec-7 has different affinities toward the two structurally close sialylated serogroups Y and W. Interestingly, we did not observe binding to the tested C-type lectins and to the other tested Siglecs.

Consistent with ELISA results, concentration-dependent reductions in Siglec-7 fluorescence intensity upon binding to Men-Y CPS and oligomers with varying numbers of repeating units were used to monitor the interactions. Therefore, the equilibrium affinity constant (*K*_D_) was determined using a nonlinear regression analysis.^34^ Fluorescence titration of increasing amounts of glycan into a fixed protein concentration showed that the tryptophane residues of Siglec-7 were quenched by ligand addition, thus proving evidence of the complex formation.^35^ The interpolation of the fluorescence data provided similar *K*_D_ values all in the low micromolar range (Figure 1B).

### Men-Y Binds to Siglec-7: NMR and Computational Studies

#### Analysis of the Interaction between Siglec-7 and Men-Y 2-Mer

Following the isolation of oligomers with different numbers of repeating units (Figure S1), binding studies with Siglec-7 commenced with the two-unit oligomer (2-mer). Therefore, the binding features of Siglec-7 and Men-Y 2-Mer were unveiled through a combination of NMR and computational tools. Ligand-based NMR experiments were conducted with the full extracellular domain (FED) of Siglec-7 produced in HEK293S cells. STD NMR analysis^36,37^ (Figures 2 and S2; in Figures S3–S4 the fraction partially acetylated at O9 of Sia) confirmed the binding and allowed us to map the interacting epitope of Men-Y 2-Mer when accommodated into the protein binding pocket (Figure 2).

**Figure 2:**
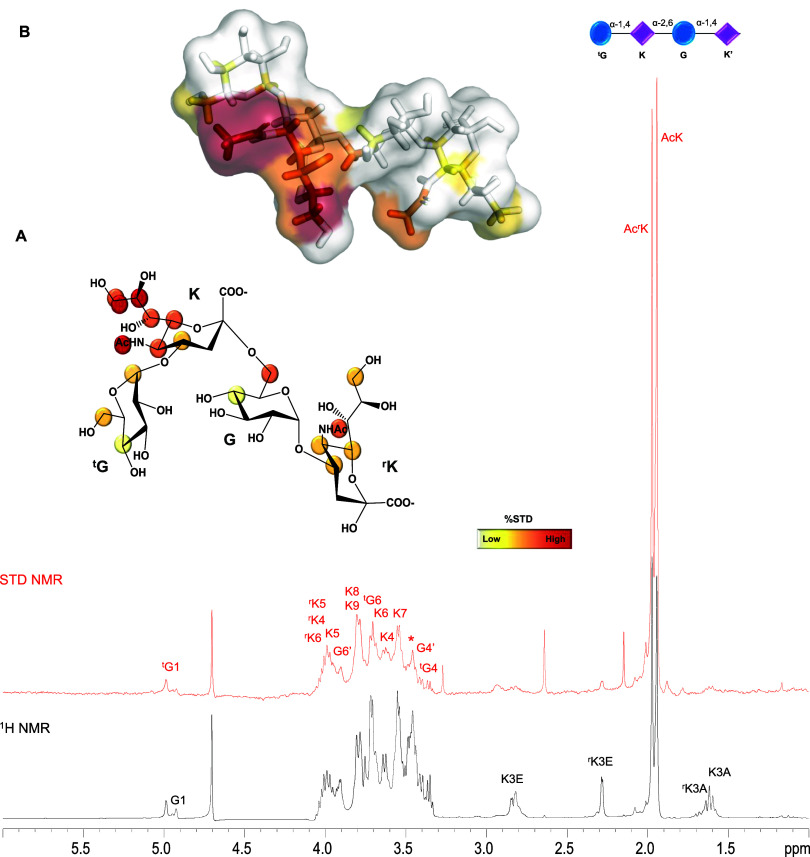
Binding studies of Men-Y 2-Mer (no acetylation at position 9 of Sia) in the interaction with Siglec-7. (A) STD NMR analysis of Siglec-7 and Men-Y 2-Mer with the ligand epitope mapping calculated by (*I*_0_ – *I*_sat_)/I_0_, where (*I*_0_ – *I*_sat_) was the signal intensity in the STD NMR spectrum (red) and *I*_0_ was the peak intensity of the off-resonance spectrum (black). The highest signal was set to 100% and the other protons were normalized accordingly. (B) Bioactive conformation of Men-Y 2-Mer as obtained by NMR; the ligand surface was colored according to the STD effects as well as protons of the structure of Men-Y 2-Mer.

The critical role of sialic acid (Neu5Ac) in Siglec-7 binding was elucidated. In particular, the internal sialic acid residue, indicated as **K**, emerged as the key determinant, displaying significantly high relative STD percentages within the binding epitope. The highest STD enhancements were set at 100%, and the other values were normalized accordingly; strong STD effects were attributed to the glycerol chain of internal Sia **K**. Discrete STD effects were observed for ring protons of both Sias (internal **K** and reducing ^r^ **K**) as well as for protons at position 6 of the internal Glc (**G**). Glucose residues also participated in the binding interaction, albeit to a lesser degree. Indeed, a lower contribution to the binding was furnished by H1 and H6 of the terminal Glc (^**t**^ **G**). The substantial overlap of resonances arising from other glucose residue protons precluded detailed epitope mapping for these sugar units; still, small STD signals were observed for the isolated H4 of ^t^ **G** and **G** (Figures 2 and S3). Therefore, internal Sia residue **K** drove the binding of Men-Y 2-Mer with Siglec-7, and Sia moiety ^r^ **K** also established important interactions. Significantly, no STD contributions were detected from the O-acetylated protons at O9 (Figure S3A). This finding suggests that the ninth position of the glycerol chain of internal Sia is recognized by Siglec-7 exclusively when it is not acetylated. In addition, rather than enhancing interaction with Siglec-7, acetylation at O9 of Sia displaces the Sia glycerol chain from the binding site. The conformational behavior of Men-Y CPS was explored in both free and bound states by NMR combined with computational studies.^38^ The flexibility of α-(2,6)-sialoglycans depends on the torsion angles around the Neu5Ac-α-(2-6)-Glc glycosidic linkage, namely, φ (C1–C2–O–C6′), ψ (C2–O–C6′–C5′), and ω (O6–C6′–C5′–O5′), where this last torsion provides further rotational freedom to the ligand (Figure S4A). Previous studies and energy maps (Figure S4B) showed that φ could explore two predominant torsion values of −60 and 180°, while ψ assumes almost stably a value of 180°.^39^ On the other hand, the ω torsion displays the highest flexibility, potentially spanning three different values corresponding to −60°/60°/180° (*gg*/*gt*/*tg*)^14^ (Figure S4). The multiplicity of the diastereotopic protons at position 6 of glucose **G** together with the vicinal coupling constant between H5 and H6 (^3^ *J*_5,6_< 3 Hz) proved that the preferred ω value adopted in solution corresponded to the *gg* rotamer (ω at −60°). We thoroughly investigated the behavior of the oligomers when acetylated at the O9 of Sia, showing how the acetylation did not induce significant conformational changes in the glycan chain (Figure S5). To evaluate the conformational behavior of Men-Y CPS in the bound state, trNOESY experiments (Figure S4C) combined with MD simulations in explicit solvent were performed (Figures S5 and S6, respectively).

The NOE contacts between the anomeric proton of glucose with the diastereotopic H3 protons of the corresponding linked sialic acid confirmed the *exo-syn* conformation of the glycosidic linkage (φ around Glc-(1→4)-αNeuNAc of −60°) (Figure S4C).^40^ Binding and conformational features were further explored with computational studies; 2-Mer was built in GLYCAM^41^ and modeled into the binding site of Siglec-7 (Figures S3–S5) based on the Sia orientation in the crystal structure of the GT1b analogue bound to Siglec-7 (PDB: 2HRL)^42^ used as a structural template. According to NMR results, the ω torsion angle was restrained at −60° (*gg*), while all of the other torsion angles were sampled along the simulation. MD analysis revealed that φ around αNeuNAc-(2→6)-αGlc mainly adopted a value around −60°, occasionally sampling at 180°, while the ψ torsion remained consistently constant at 180° along the MD simulation (Figure S6A). The stability of the complex was demonstrated by root-mean-square deviation (RMSD) analysis of the MD simulation (Figure S6B), confirming the stable positioning of the ligand in the Siglec-7 binding pocket. Mapping the interactions between 2-Mer and Siglec-7 allowed the molecular description of binding in the 3D complex (Figure 3).

**Figure 3:**
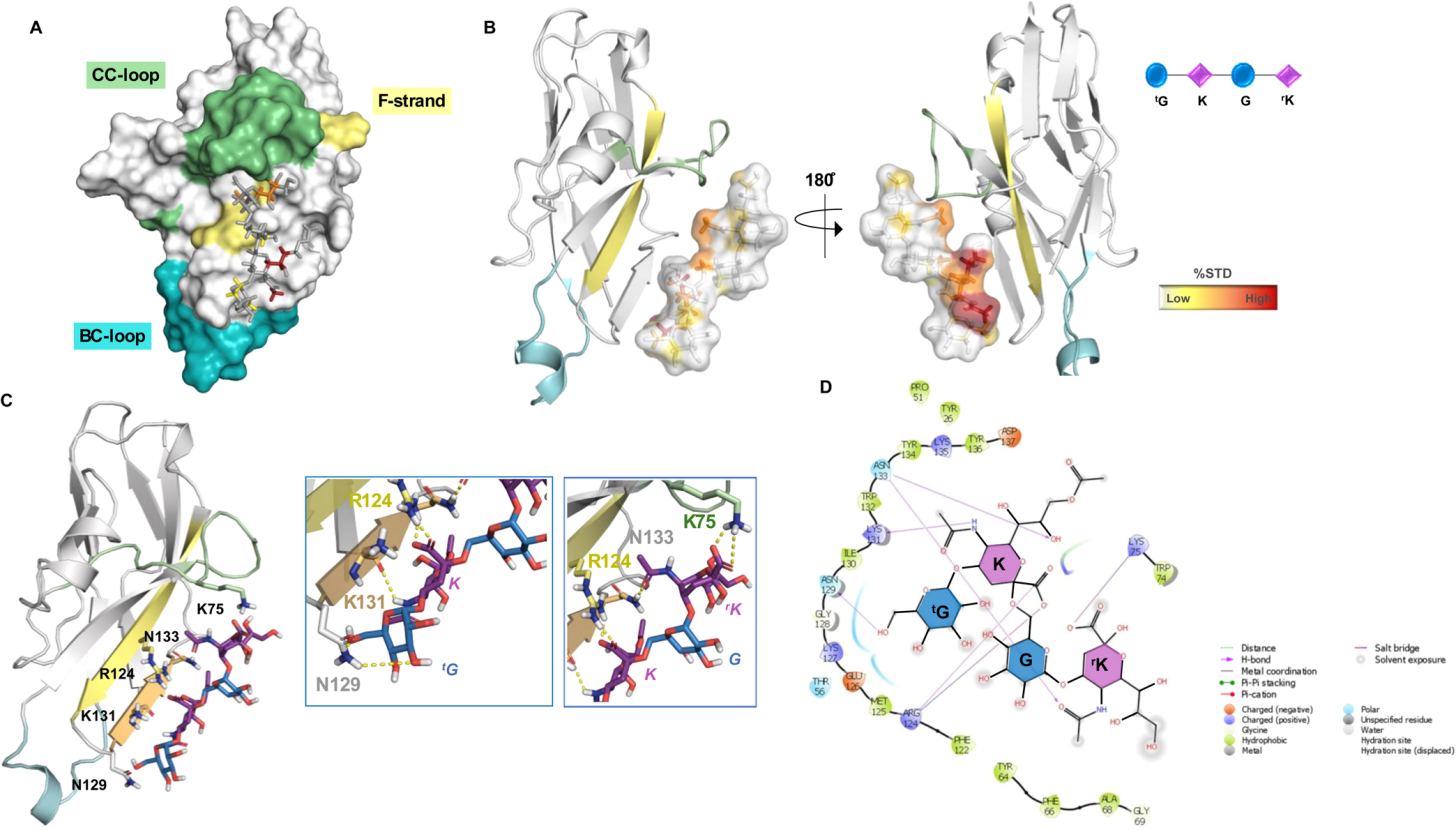
3D model of Siglec-7 and 2-Mer acetylated at position 9 of internal sialic acid. (A) 3D complex with the protein surface colored by strand and loops, as indicated in the figure. (B) 3D views of the Siglec-7/Men-Y 2-Mer complex: the strands involved in the recognition are colored by type: F—β strand in yellow, BC′—loop in cyan, and CC′—loop in green. The ligand surface was colored according to the STD edit code. (C) Different views highlighting the H-bonds monitored by MD simulation are shown. Ligand 2-Mer is represented following the SNFG color code. (D) 2D plot of the interactions occurring at Siglec-7 and 2-Mer’s from the Men-Y interface.

Analysis of key contacts revealed mainly polar, electrostatic and hydrogen bond interactions, most of them retained during the MD simulation. This network of polar interactions involved highly conserved residues located on the F-β strand, surrounding the canonical Arg124, and on the G-β strand, in which typical amino acids of Siglec-7 binding pocket, such as Lys131 and Asn133, were located (Figure 3C,D). In detail, the highly conserved Arg124 established a salt bridge with the carboxylate group of the internal Neu5Ac (**K**) residue via its guanidinium group.^43–46^ In line with STD NMR, the Neu5Ac (**K**) glycerol moiety was involved in hydrogen bonds between the 8-OH′ position with the Asn133 lateral chain; in addition, the *N*-acetyl group of Neu5Ac (**K**) established a stable hydrogen bond with Lys131. Notably, these two residues, Asn133 and Lys131, located on the G-strand as mentioned above, are highly conserved and assist the accommodation of Men-Y CPS in the binding site. Conversely, the acetyl group at position 9 of **K** did not interact with Siglec-7 and remained solvent-exposed throughout the entire MD simulation (Figure 3C,D). Almost all of the protons belonging to both internal (**G**) and terminal Glc unit (^**t**^ **G**) were solvent-exposed, except those at positions 1 and 6 of ^t^ **G** and **G** residues, respectively, and the hydroxyl group O6 of ^t^ **G** that established a single interaction with the side chain of Asn129 inside the binding pocket of the protein. The model further predicted H-bonds between Lys75 and Asn133 with the carboxyl and acetyl groups of ^**r**^ **K**. Therefore, MD analysis aligned with the findings from STD NMR (Figure 3B), emphasizing the primary involvement of Neu5Ac **K** in the recognition and binding event, and showing the minor role of reducing Neu5Ac (^**r**^ **K**) and Glc (**G**) in the interaction. Remarkably, the absence of acetylation allowed the OH to establish a H-bond with Asn133, which was already involved in the interaction with the OH at position 8 (Figure S7B). By integrating the analysis of contacts observed in MD simulations, the epitope mapping, and the bioactive conformation derived from NMR data, a comprehensive 3D depiction of the Siglec-7 and 2-Mer from Men-Y interaction was attained (Figure 3).

#### Analysis of the Interaction between Siglec-7 and Longer Oligomers

The molecular details of the interaction between Siglec-7 and Men-Y CPS were also revealed via protein-based NMR and computational studies. Aliquots of Men-Y 4-Mer were sequentially added to [U–^15^N] labeled Siglec-7 CRD, and ^1^H–^15^N-HSQC NMR spectra were acquired.^23^ As expected, during the NMR titration, some protein signals experienced chemical shift perturbation (CSP) and/or decrease in signal intensity (Figures 4, S8, and S9).^47^ In Figure 4, the highest CSPs were observed for residues of the canonical binding site, such as Glu126, Lys131, Trp132, Asn133, and Lys135^GG′^ and, to a lesser extent, Tyr136^GG′^. In proximity of the binding site, Val61, Ile72^CC′^, and Phe123 also experienced CSPs. Similarly, residues located in the canonical site, including Glu126, Lys131, and especially Asn133, experienced the strongest decrease in signal intensity (Figure 4). The vicinal Tyr134^GG′^as well as Asn55^BC′^, Lys75^CC′^, and residues Asp59, Glu90, Arg94, and Phe95 were also perturbed by intensity decreases but to a lesser extent. These variations confirmed the binding and defined the primary binding site of Siglec-7 with Men-Y. Ligand-based NMR studies demonstrated that 4-Mer exhibited a bioactive conformation and binding epitope comparable to those of the previously reported 2-Mer (Figure S7C).

**Figure 4:**
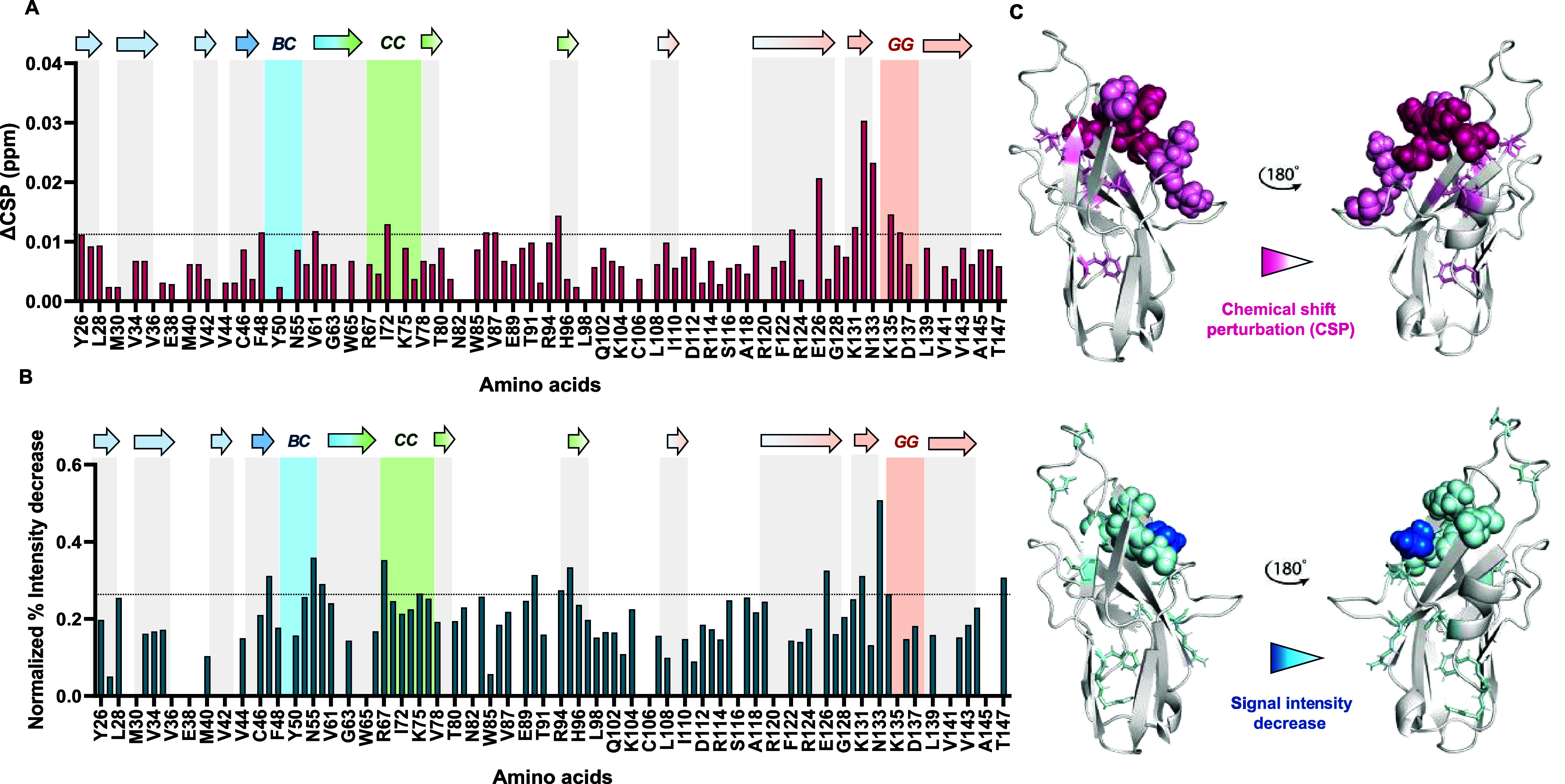
Protein-based NMR titration of ^15^N Siglec-7 with 4-Mer Men-Y. (A) Diagram of the chemical shift perturbation (CSP) of aa of 100 μM Siglec-7 in the presence of large excess of Men-Y. The CSP effects were evaluated with the formula $\mathrm{CSP}=\frac{1}{2}\sqrt{{{\Delta \delta }_{H}}^{2}+{\left({\Delta \delta }_{N}/5\right)}^{2}}$ and the threshold (dotted line) was set based on the second standard deviation. The residues experiencing the CSP effects above the threshold (highlighted in pink on the 3D structures) were Y26, F48, V61, I72, A86, V87, F95, F123, E126, K131, W132, N133, K135, and Y136. Among these, E126, K131, W132, and N133 exhibited the strongest CSP effects and are shown in a more intense pink. (B) Diagram of the % intensity decrease of aa of 100 μM Siglec-7 in the presence of a large excess of Men-Y. The % intensity decrease effects were evaluated from the variation of chemical shift heights between the protein in apo form and bound to CPS, then normalized to the maximum value. The threshold (dotted line) was set based on the standard deviation. The residues experiencing the signal’s intensity decrease higher than the threshold set (highlighted in light cyan on the 3D structures) were S47, N55, D59, R67, K75, E90, R94, F95, E126, K131, N133, Y134, and T147. N133 experienced the highest intensity decrease and was colored in blue. (C) 3D structures of Siglec-7 (PDB 2HRL) evidencing the most perturbed amino acids in CSP (pink spheres) and signal intensity decrease (blue spheres). Amino acids located farther from the binding site are shown as colored sticks, while residues closer to the binding site, where the key R124 is located, are displayed as spheres.

Similarly, titration of Siglec-7 with Men-Y CPS revealed numerous CSPs and decreases in signal intensity (Figure S9). As observed during the oligomer titration (Figure 4), Lys131, Trp132, and Lys136^GG′^ experienced the strongest CSPs, followed by Tyr26 and Phe95, while Asn55 and Lys131 experienced a decrease in their signal intensity; instead, other residues were affected only in the presence of Men-Y CPS. In particular, Ser27, Phe48^BC^, Asn55^BC^, Arg92, His96, Asp137^GG′^, and Gln138^GG′^ experienced CSPs, while Ala76, Asn105, Leu108, Ser116, and Arg124 were affected by a decrease in their signal intensity. Moreover, Asn55 and Arg92 experienced both types of variations (Figure S9). Therefore, residues located in the canonical binding site, such as Arg124 and Lys131, together with residues in the BC and CC′ loops, including Asn55^BC^ and Ala76^CC′^, experienced a decrease in signal intensity (Figure S9), suggesting their involvement in the interaction with Men-Y CPS. Notably, Ala76 of the CC′ loop likely participate in the binding, since it disappeared upon binding giving the highest variation (Figure S9). CSPs were particularly noticeable in signals located close to Arg124, including Tyr26 and Ser27. Furthermore, we observed that several signals affected by CSP were located near and on the GG′ loop, including Lys131, Trp132, Tyr136^GG′^ Asp137^GG′^, and Gln138^GG′^. Indeed, these amino acids are generally involved in establishing hydrophobic interactions with the sialic acid residue, stabilizing the complex. Other CSP effects were attributed to Arg92, Phe95, and His96, which might form a pocket where the extended glycan chain could be accommodated, suggesting an extended binding mode for this longer ligand (Figure 5).

**Figure 5:**
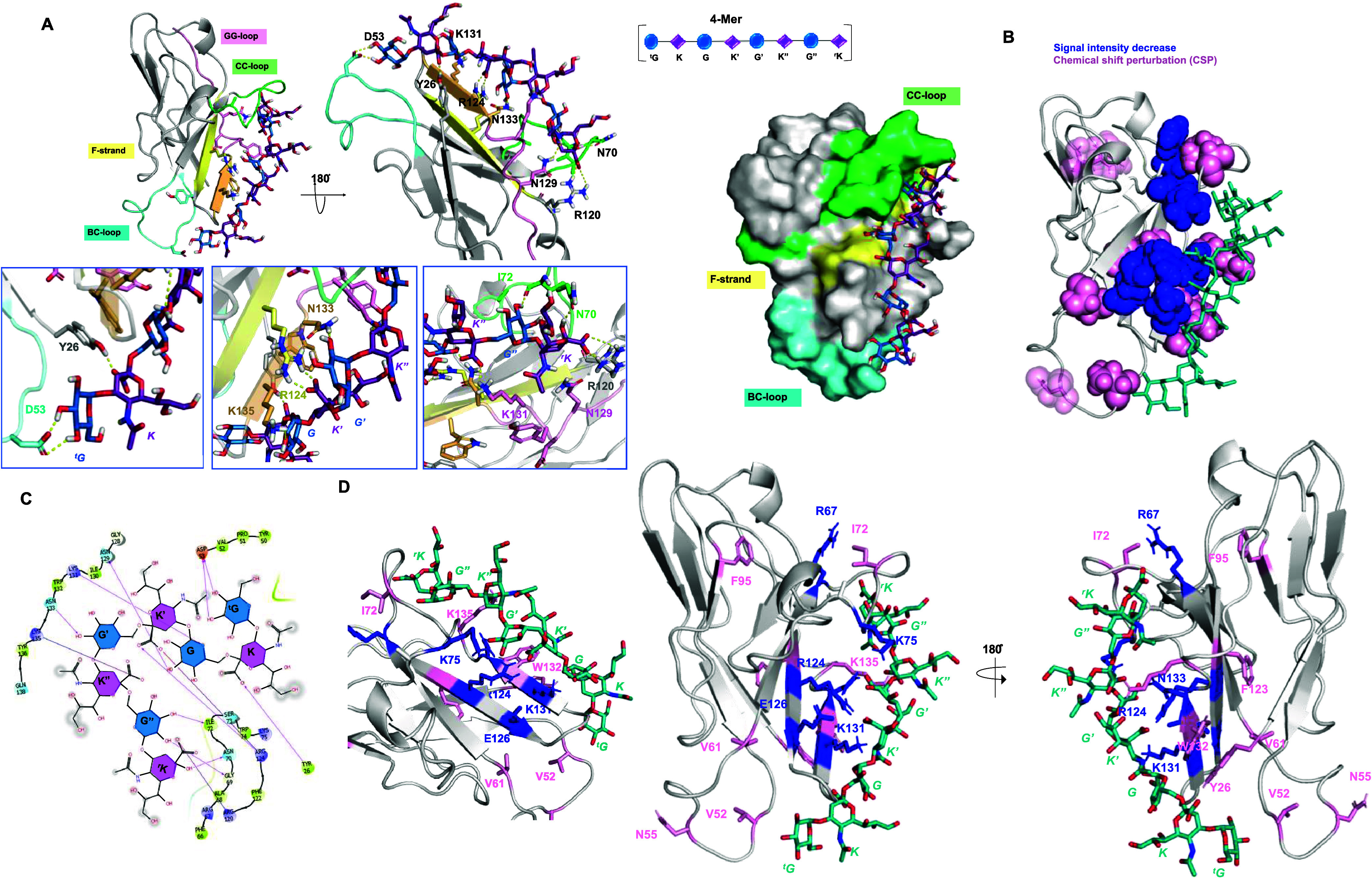
3D model of the Siglec-7 and Men-Y 4-Mer complex. **(A)** Different views highlighting the H-bonds monitored by MD simulation were shown. The strands involved in the recognition are colored by type: F—β strand in yellow, BC—loop in cyan, CC′—loop in green, and GG—loop in pink. Ligand 4-Mer is represented following the SNFG color code. The 3D surface model of the Siglec-7–4-Mer complex is also presented. (B) 3D views of Siglec-7 in complex with 4-Mer (teal), highlighting the amino acids according to signal intensity decrease (blue) and CSPs (pink). (C) 2D plot of the interactions occurring at the Siglec-7 and Men-Y 4-Mer interface. (D) 3D views of Siglec-7 in complex with 4-Mer (teal), highlighting the interactions between the amino acids shown as sticks and colored according to signal intensity decrease (blue) and CSPs (pink).

These data were further supported by the computational studies performed with the 4-Mer, built and modeled into the binding pocket of Siglec-7 (PDB: 2HRL). Given that experimental data indicated a low 20% acetylation rate at the C-9 position of Neu5Ac and considering that 2-Mer studies showed no significant impact of acetylation on the entire recognition and binding, we opted to use the deacetylated structure of 4-Mer for computational analysis. As depicted in Figure 5A, the Neu5Ac residue K′ was the primary recognition site for the protein, engaging in a specific interaction between its carboxylate group and the guanidinium of the conserved Arg124, situated on the F-β strand of the Siglec-7 binding pocket. In addition, the carboxylate moiety of **K″** established a hydrogen bond with Lys135, while **G** and **G′** glucose residues established isolated H-bonds with conserved aa residues of the G-strand, such as Asn133 and Lys131 (Figure 4B,C), favoring the accommodation of 4-Mer into the binding cavity. Moreover, Neu5Ac (**K**) and the terminal glucose (^**t**^ **G**) residues established further contacts with the BC loop to further accommodate the ligand inside the binding pocket, establishing contacts with Tyr26 and Asp53, respectively, in agreement to protein-based NMR studies (Figure 5D). Some other interactions were predicted, including hydrogen bonds formed with some CC′ loop residues. The carbonyl lateral chain of Asn70 with the reducing hydroxyl of ^**r**^ **K**, and Arg120 with ^**r**^ **K** carboxylate group helped to stabilize the ligand in the binding pocket. Side interactions were also observed, such as those between the **G″** glucose unit with Ile72 and Gly69. Notably, a comparable binding mode was predicted for both 2-Mer and 4-Mer (Figure S10). In both cases, internal sialic acid (K′) formed a critical salt bridge with Arg124. However, a slight spatial displacement of 4-Mer relative to 2-Mer was observed, resulting in subtle alterations at the protein–ligand interface, which is important for the accommodation of the longer glycan chain.

### Evaluation of Immunological Response Triggered by Men-Y CPS

A preliminary evaluation of Men-Y CPS’s ability to modulate the host immune response was conducted by investigating its effects on U937-derived macrophages (Figure 6A), which express Siglec-7 on their surface,^48^ and THP-1-derived macrophages (Figure 6B) as a control, exhibiting lower Siglec-7 expression.^49^ The inflammatory response of macrophages is characterized by an increased secretion of many proinflammatory and chemotactic cytokines; we chose to analyze *TNF*α and *IL-8* as proinflammatory cytokines and *CXCL10* expression as a chemotactic cytokine. The treatment of THP-1-derived macrophages induced a weak but significant proinflammatory and chemotactic response to 0.1 μg/mL of Men-Y treatment as evidenced by *IL-8* and *CXCL10* upregulation, respectively (Figure 6B). This response was even more amplified after treatment with 1 μg/mL Men-Y CPS, as displayed by the very strong upregulation of the three genes (Figure 6B), especially for *CXCL10*. On the other hand, U937-derived macrophages appeared indifferent to both doses of Men-Y CPS, as the expression of *TNFa* and *IL-8* cytokines and *CXCL10* chemokine was comparable to nontreated cells (Figure 6A), indicating that this capsular polysaccharide failed to activate the immune response, likely due in part to its interaction with Siglec-7, which inhibits immune responses via its ITIM domain. This evidence was further confirmed by treating both U937- and THP-1-derived macrophages with E. coli LPS. Both cell types could efficiently induce a proinflammatory response, even at the lowest LPS concentration (0.1 μg/mL). The response was further amplified at 1 μg/mL.

**Figure 6:**
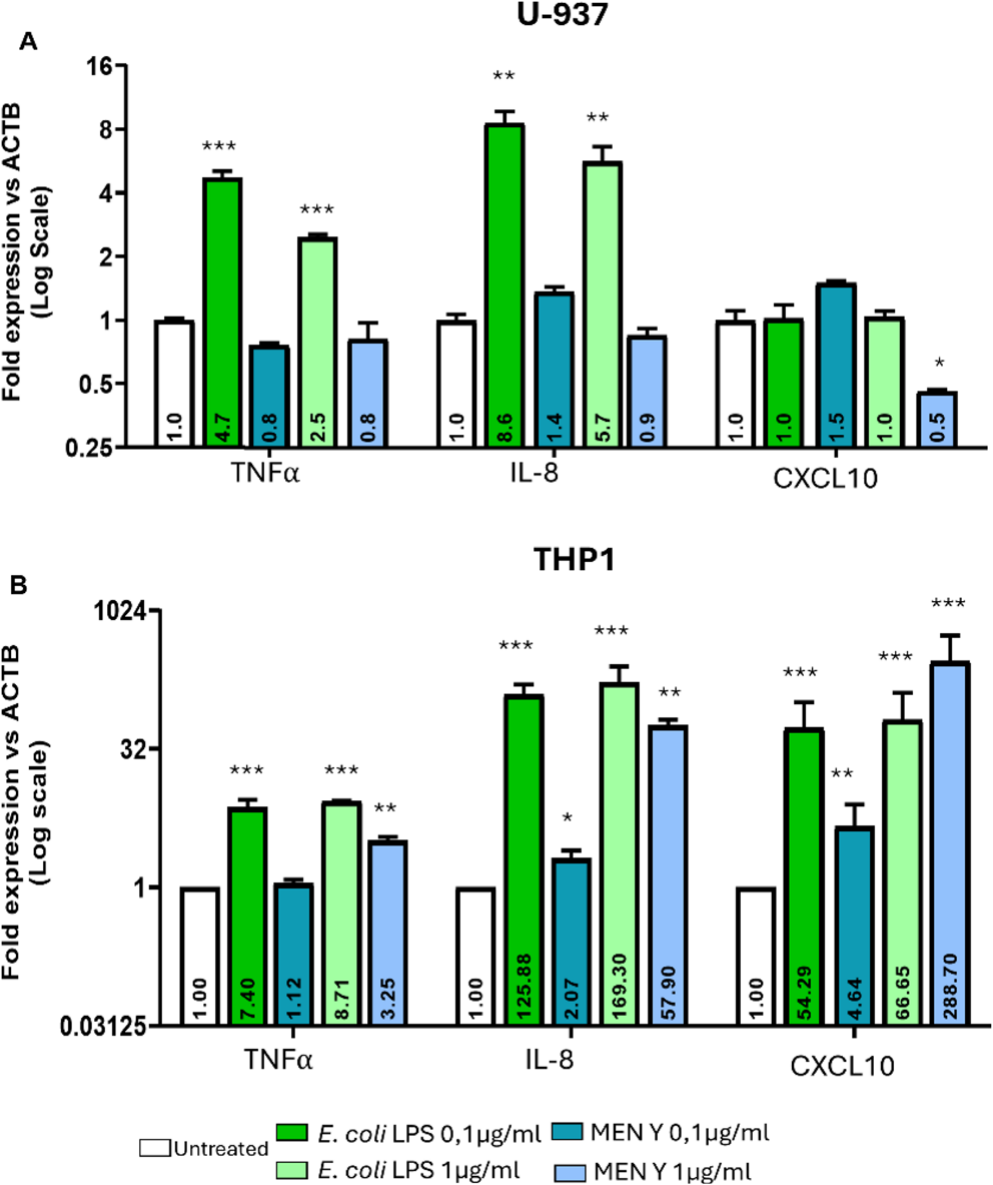
Semiquantitative analysis on the production of the proinflammatory cytokines from U937 and THP1 cell lines after stimulation with Men-Y CPS. E. coli LPS was used as a positive control.

## Conclusions

*Nm* capsular polysaccharides are essential for virulence and are key components of glycoconjugate vaccines. Sialylated CPS might enable immune evasion and modulation by interacting with inhibitory Siglec receptors such as Siglec-7. Understanding this interaction can further elucidate *Nm* immune evasion strategies and inform the development of targeted interventions. To this aim, the comprehensive analysis developed in this work provides a full view of the specific recognition of sialylated CPS from serogroup Y (Men-Y) by Siglec-7.

Our studies revealed that Men-Y capsular polysaccharide selectively bound Siglec-7 compared to other tested human immune lectins and in contrast to Men-W CPS. Indeed, the single C4 hexose stereochemistry difference (Gal vs Glc) with respect to Men-W results in significantly different Siglec-7 binding, with Men-Y showing higher affinity. Although NMR and modeling indicate partial H4-Glc involvement in the interaction, the binding difference likely arises from distinct glycan chain conformations of the two CPS, therefore, influencing pocket accommodation. To dissect the binding features and unravel the molecular basis of recognition, a multidisciplinary approach was used, combining ELISA, fluorescence titration studies, ligand- and protein-based NMR, *in silico* analysis, and biological assays. Preferential binding, key interactions, conformational properties and spatial arrangement each contribute to the complexity and specificity of this host–pathogen interaction. Ligand-based NMR spectroscopy highlighted the central role of the internal sialic acid (**K**) in the binding epitope, particularly with the *N*-acetyl group and the glycerol chain. Computational studies elucidated that the binding to Siglec-7 was primarily mediated by electrostatic and hydrogen bond interactions. Arg124, whose guanidinium group established the crucial salt bridge with Sia **K**, along with Lys131 and Asn133, emerged as key residues within the conserved Siglec-7 binding site. Additional interactions helped to anchor the ligand to the protein binding site, like H-bonds of Asn133 with **K**8 and of Lys131 of the GG′ loop with the NH at position 5 of **K**, in complete accordance with the STD NMR results. Notably, as observed in protein-based NMR experiments, both Asn133 and Lys131 were affected by signal intensity decreases and CSPs; these interactions involving the internal sialic acid were in good accordance with those described in the X-ray complex (PDB: 2df3, Figure S11). Additionally, in our complex, carboxylate and acetyl groups of the reducing sialic acid (^r^ **K**) established H-bonds with Lys75^CC′^ and Asn133, respectively, while proton H6 of terminal galactose made a H-bond with Asn129. Moreover, we demonstrated via STD NMR that the presence of the acetyl group at position 9′ of the Neu5Ac residue did not affect the binding; furthermore, in the predicted complexes, this position was solvent-exposed when acetylated. The GG′ loop was then involved in the binding mainly with Lys131, a residue affected by both signal intensity decrease and CSP, and also with hydrophobic amino acids Trp132 and Tyr136, both influenced by CSP effects. Overall, the residues Tyr26, Lys131, Trp132, and Tyr136^GG′^ together with Phe48, Asp137^GG′^, and Gln138^GG′^ and the key Arg124, were affected by the binding of Men-Y CPS, defined as the main binding site where the oligomers could accommodate. Regardless of the glycan length, the binding affinity was comparable, making Sia and internal Glc crucial in the recognition. Lastly, *Nm* Men-Y CPS was also preliminarily evaluated for its ability to induce immune modulation, and while it induced a significant proinflammatory and chemotactic response in THP-1-derived cells, it did not affect U937-derived macrophages, which express a higher amount of Siglec-7.

## Supplementary Material



## Notes

^1^H and HSQC NMR spectra of depolymerized CPS; binding studies on 2-Mer partially acetylated; further computational studies and NMR binding studies on acetylated 2-Mer and 4-Mer; zoom of ^15^N titration experiments, on 4-Mer and on CPS; and further details of 3D complexes (PDF)

## Abbreviations

Siglecs: sialic-acid-binding immunoglobulin-like lectins
NK: natural killer
CPS: capsular polysaccharide
Men-Y: Neisseria meningitidis serogroup Y
ELISAs: enzyme-linked immunosorbent assays
NMR: nuclear magnetic resonance
Nm: Neisseria meningitidis
Sia: sialic acid
CSP: chemical shift perturbation
CRD: carbohydrate recognition domain
MD: molecular dynamics
ITIM: immunoreceptor tyrosine-based inhibition motif
STD: saturation transfer difference
FED: full extracellular domain
RMSD: root-mean-square deviation
NOE: nuclear Overhauser effect
