# Supplementary material for: Molecular Basis of Siglec‑7 Recognition by Neisseria meningitidis Serogroup Y CPS: Implications for Immune Evasion
Source: JACS Au. 2025 Apr 30;5(5):2257–69. doi: 10.1021/jacsau.5c00214 (PMC12117448; doi:10.1021/jacsau.5c00214)

Supplementary material for:

Molecular Basis of Siglec-7 Recognition by *Neisseria meningitidis* Serogroup Y CPS: Implications for Immune Evasion

Cristina Di Carluccio[a], Tania Gerpe Amor[a], Maria Pia Lenza[a], Alessandro Antonio Masi[a], Celeste Abreu[b], Viviana Longo[c], Francesco Albano[c], Ferran Nieto-Fabregat[a], Paola Salvatore[d], Geppino Falco[c], Darielys Santana-Medero[e], Marco Fragai[f], Yvette van Kooyk[g], Antonio Molinaro[a], Yury Valdes-Balbin[e], Ondřej Vaněk[b], Vicente Verez-Bencomo[e], Roberta Marchetti[a], Fabrizio Chiodo[g,h], Alba Silipo[a*]

a. Department of Chemical Sciences, University of Naples Federico II, Via Cinthia 4, 80126, Naples, Italy; silipo@unina.it
b. Department of Biochemistry, Faculty of Science, Charles University, Hlavova 2030/8, 12800, Prague, Czech Republic
c. Stem Cell Biology Laboratory, Department of Biology, University of Naples Federico II, Naples, Italy
d. Department of Molecular Medicine and Medical Biotechnology, University of Naples Federico II, Via Pansini, 5, 80131, Naples, Italy.
e. Finlay Vaccine Institute. 21st Ave. N◦ 19810 between 198 and 200 St, Atabey, Playa, Havana, Cuba
f. Magnetic Resonance Centre (CERM), CIRMMP and Department of Chemistry "Ugo Schiff", University of Florence, 50019, Sesto Fiorentino, Italy
g. Department of Molecular Cell Biology and Immunology, Amsterdam UMC, Vrije Universiteit Amsterdam, Amsterdam, 1081 HV, The Netherlands.
h. Institute of Biomolecular Chemistry, National Research Council (CNR), Via Campi Flegrei, 34, 80078, Pozzuoli, Naples, Italy

This file includes:

Supporting figures S1-S11: ^{1}H and HSQC NMR spectra of depolymerized CPS. Binding studies on 2-Mer partially acetylated. Further computational studies and NMR binding studies on acetylated 2-Mer and on 4-Mer. zoom of ^{15}N titration experiments, on 4-Mer and on CPS. Further details of 3D complexes

Table S1 with structure of representative *Neisseria meningitidis* serogroups;

Supporting Figure Legends

Table S1. Most infectious serogroups from *Neisseria meningitidis* are divided into ten groups, where four are sialylated.

Figure S1. ^{1}H-NMR of Men-Y CPS and oligomers obtained after mild acid hydrolysis.

Figure S2. A. ^{1}H-^{13}C-HSQC spectrum of 2-Mer from Men-Y with partial acetylation at position 9 in its free state (600 MHz, D_2O). K8 (Ac) and K7(Ac) indicate the protons at position 8 and 9 of sialic acid when ligand is partially acetylated. **B.** ^{1}H-^{13}C-HSQC spectrum of 2-Mer from Men-Y with no acetylation at position 9 in its free state (600 MHz, D_2O).

Figure S3. Binding studies of 2-Mer with partial acetylation at position 9 in complex with Siglec-7. **A**. STD NMR analysis of Siglec-7 and Men-Y 2-Mer with the ligand epitope mapping calculated by $(I_0 - I_{sat})/I_0$, where $(I_0 - I_{sat})$ was the signal intensity in the STD-NMR spectrum (black) and I_0 was the peak intensity of the off-resonance spectrum (green). The highest signal was set to 100% and the other protons were normalized accordingly. **B.** Bioactive conformation of Men-Y 2-Mer as obtained by NMR; the ligand surface was colored according to the STD effects as well as protons of the structure of Men-Y 2-Mer.

Figure S4. A. Representation of the three staggered rotamers from the torsional angles described for the linkage α-Neu5Ac-(2,6)-α-Glc: φ, ψ, and ω, where the ω have as main values -60, 60°, and 180° – namely *gg*, *gt*, and *tg*, respectively, around the C5-C6 bond. **B.** Adiabatic energy maps of the two different glycosidic linkages present in the serogroup Men-Y, illustrating the φ, ψ, and ω torsions of the Sia-Glc linkage; and the φ and ψ torsions or the Glc-Sia bond. **C.** tr-NOESY of Men-Y in its bound state with Siglec-7 using a mixing time of 400 ms at 600 MHz. On the left, most representative clusters from the MD simulation of 2-Mer Men-Y are represented as sticks in agreement with tr-NOESY bioactive conformation.

Figure S5. Different acetylation patterns for Men-Y, MD simulation in the free state. The behavior of φ, ψ, and ω torsion angles along MD simulations of 2-Mer are reported. The stability of the ligand is represented by the RMSD, plotted in red.

Figure S6. A. Dihedral angles around glycosidic linkages of 2-Mer Men-Y monitored along MD simulation in the bound state. A restriction of ω at -60° was applied, according to NMR results. **B.** RMSD trajectory of the same simulation.

Figure S7. Superimposition between 2-Mer from Men-Y acetylated at O9 of **K** (shown as pale-green sticks) and de-*O*-acetylated Men-Y (shown as light-orange sticks) when bound to Siglec-7. **A.** 3D view of both acetylated and not acetylated Men-Y in complex with Siglec-7, both accommodation and interaction are comparable. The glycerol chain of **K** residue points toward the solvent, showing no contribution to the recognition process, as highlighted in a dark-orange circle. **B.** 2D plot representation of non-acetylated (upper panel) and acetylated (lower panel) 2-Mer in complex with Siglec-7. **C**. STD NMR (left) and tr-NOESY (right) of 4-Mer with Siglec-7.

Figure S8. HSQC NMR spectra of the titration between ^{15}N Siglec-7 (100 μM) and 4-Mer Men-Y at the following protein/ligand molar ratios: 1:0 (green), 1:1 (blue), 1:10 (purple) and 1:20 (red). The CSP effects of E126, W132 and N133 were evidenced in the zoom.

Figure S9. Protein-based NMR titration of ^{15}N Siglec-7 with CPS Men-Y. **A.** Diagram of the % intensity decrease of aa of 100 μM Siglec-7 in the presence of 1 mg Men-Y oligomer. The % intensity decrease effects were evaluated from the variation of chemical shift heights between the protein in apo form and bound to CPS Men-Y, then normalized to the maximum value. The threshold (dotted line) was set based on the standard deviation. The residues experiencing the largest signal intensity decrease were Q55, R92, F95, N105, L108, S116, R124, and K131. **B.** Diagram of the chemical shift perturbation (CSP) of aa of 100 μM Siglec-7 in the presence of 1 mg CPS from Men-Y. The CSP effects were evaluated with the formula $\mathrm{CSP} = \frac{1}{2}\sqrt{\Delta\delta_H{}^2 + (\Delta\delta_N/5)^2}$ and a threshold (dotted line) was set based on the standard deviation. The residues experiencing the largest CSP were Y26, S27, F48, N55, R92, F95, H96, K131, W132, Y136, D137, and Q138. **C.** 3D structure of Siglec-7 (PDB 2HRL) evidencing the most perturbed amino acids in signal intensity decrease (blue spheres) and CSP (pink spheres).

Figure S10. A. Superimposition between 4-Mer (cyan) and 2-Mer (grey) complexed with Siglec-7. **B.** Superimposition between 4-Mer (cyan) and de-*O*-acetylated 2-Mer (yellow) complexed with Siglec-7. **C.** Superimposition between 4-Mer (cyan), 2-Mer (grey), and de-*O*-acetylated 2-Mer (yellow) complexed with Siglec-7.

Figure S11. Superimposition of Siglec-7 interacting with non-acetylated 2-Mer (yellow) proposed in this study and PDB 2df3 (salmon). The primary sialic acid is similarly recognized, but the carbonyl group at position 5 (highlighted by black circle) points far from the protein in our model with respect to the PDB (highlighted by salmon circle).

Table S1.

Serogroup	Structure of the repeating unit
**A**	→ 6)-α–D-ManNAc(3OAc)(1-PO_4 →
**B**	→ 8)-α–D-Neu5Ac(2→
**C**	→ 9)-α–D-Neu5Ac(7/8OAc)(2→
**H**	→ 4)-α–D-Gal(1 → 2)-glycerol-3-PO_4→
**L**	→ 3)-α–D-GlcNAc(1 → 3)-β-D-GlcNAc(1 → 3)-α-D-GlcNAc-1-PO_4→
**X**	→4)-α–D-GlcNAc-1-PO_4→
**Y**	→ 4)-α–D-Neu5Ac(7/9OAc)(2 → 6)-α-D-Glc-(1 →
**W135**	→ 4)-α–D-Neu5Ac(7/9OAc)(2 → 6)-α-D-Gal-(1→
**Z**	→ 4)-α–D-GalNAc(1 → 2)-glycerol-3-PO_4→
**29E**	→ 4)-α–KDO(1 → 3)-α-D-GalNAc(1→

Figure S1.

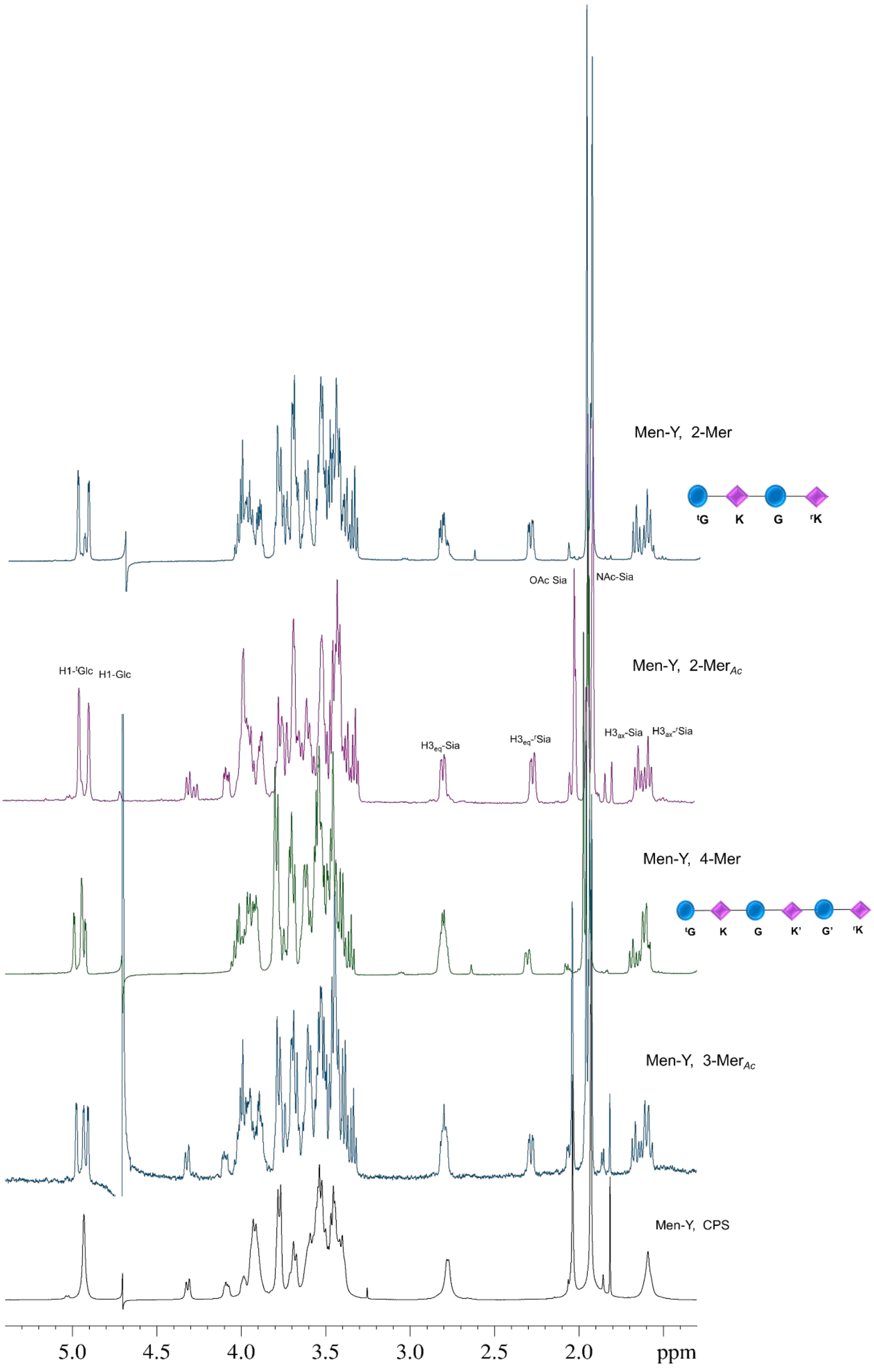
Men-Y, 2-Mer
^{t}G K G ^{r}K
OAc Sia
NAc-Sia
Men-Y, 2-Mer$_{Ac}$
H1-^{t}Glc
H1-Glc
H3$_{eq}$-Sia
H3$_{eq}$-^{r}Sia
H3$_{ax}$-Sia
H3$_{ax}$-^{r}Sia
Men-Y, 4-Mer
^{t}G K G K' G' ^{r}K
Men-Y, 3-Mer$_{Ac}$
Men-Y, CPS
5.0
4.5
4.0
3.5
3.0
2.5
2.0
ppm

Figure S2.

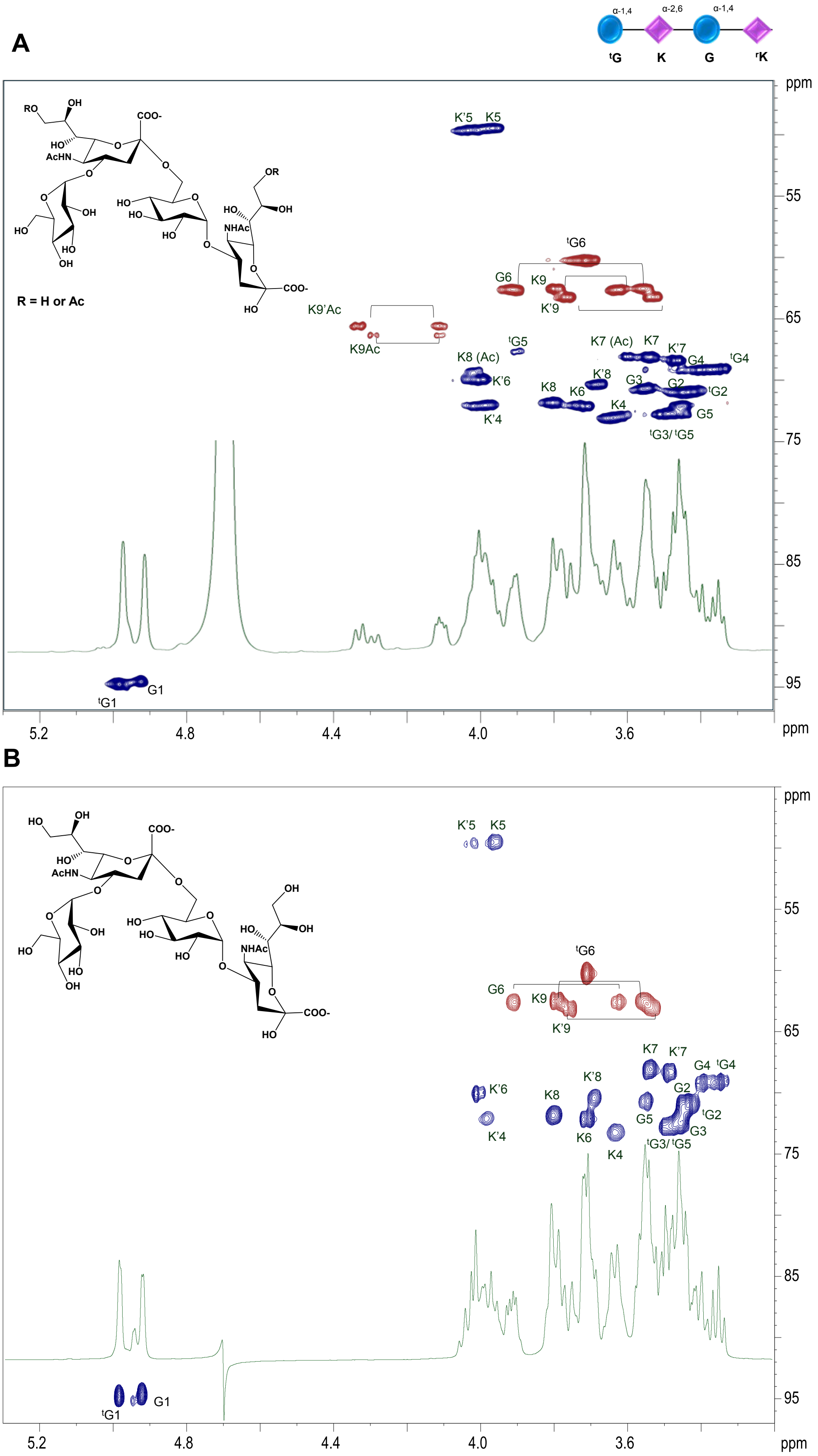

A
α-1,4
α-2,6
α-1,4
tG
K
G
rK
ppm
K'5 K5
tG6
G6
K9
K'9
K9'Ac
K9Ac
tG5
K8 (Ac)
K'6
K7 (Ac)
K7
K'7
G4
tG4
K'8
G3
G2
tG2
K8
K6
K4
K'4
G5
tG3/ tG5
R = H or Ac
G1
tG1
5.2
4.8
4.4
4.0
3.6
55
65
75
85
95
B

Figure S3

A

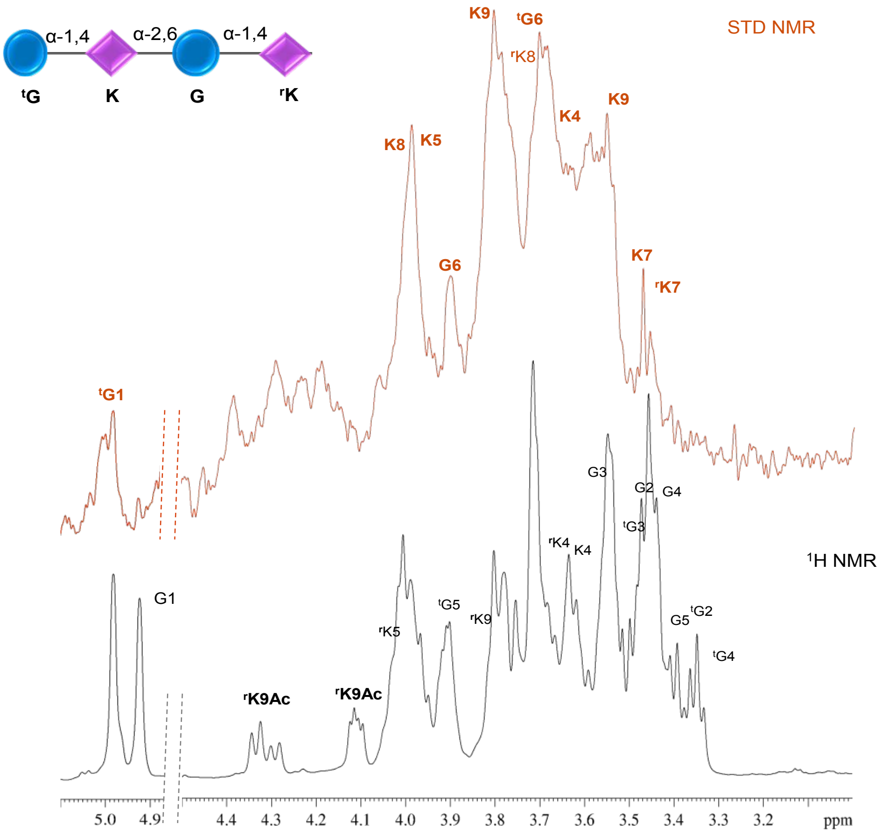

α-1,4
α-2,6
α-1,4
tG
K
G
rK
STD NMR
1H NMR
ppm

B

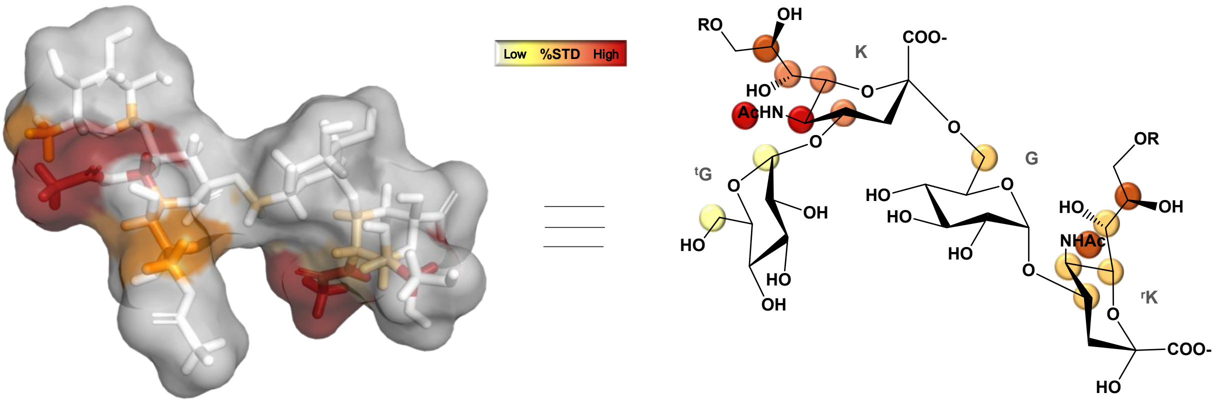

Low %STD High
RO
OH
COO-
K
HO
AcHN
O
tG
G
OR
HO
OH
NHAc
rK
COO-

Figure S4.

A

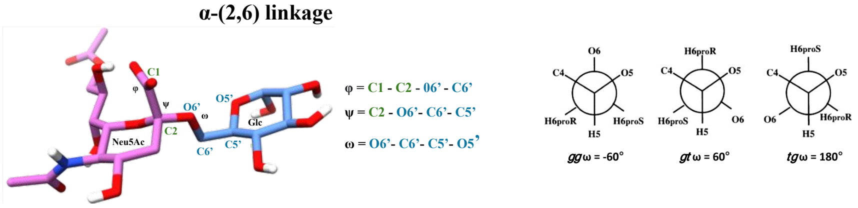

B

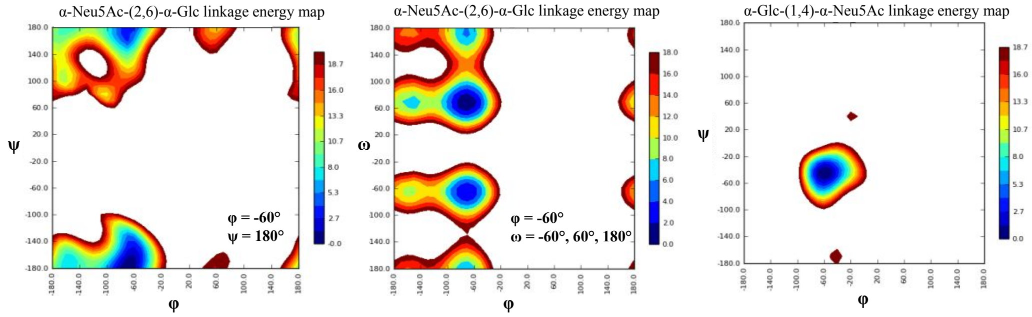

C

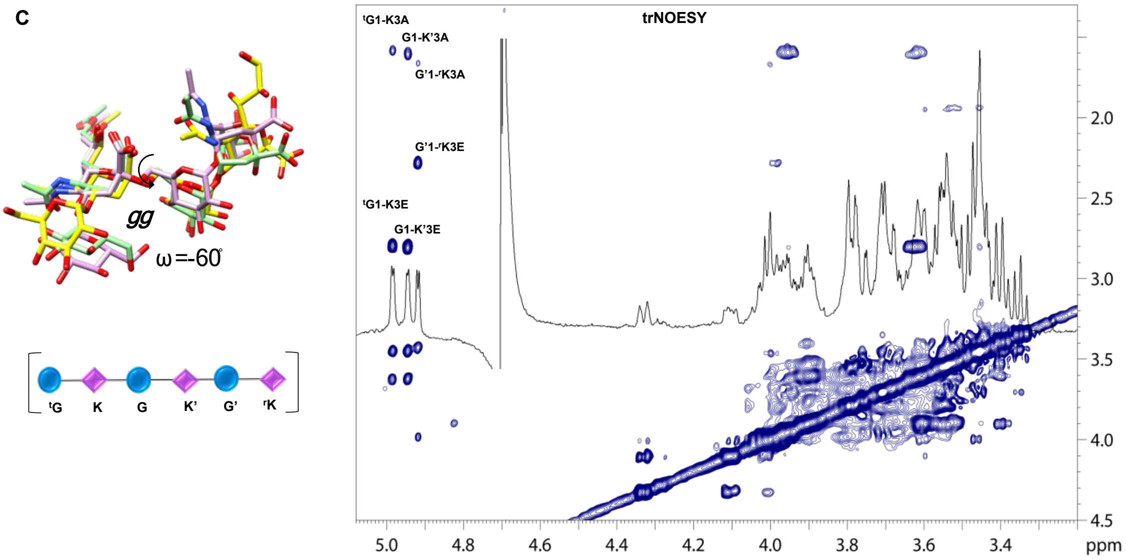

Figure S5.

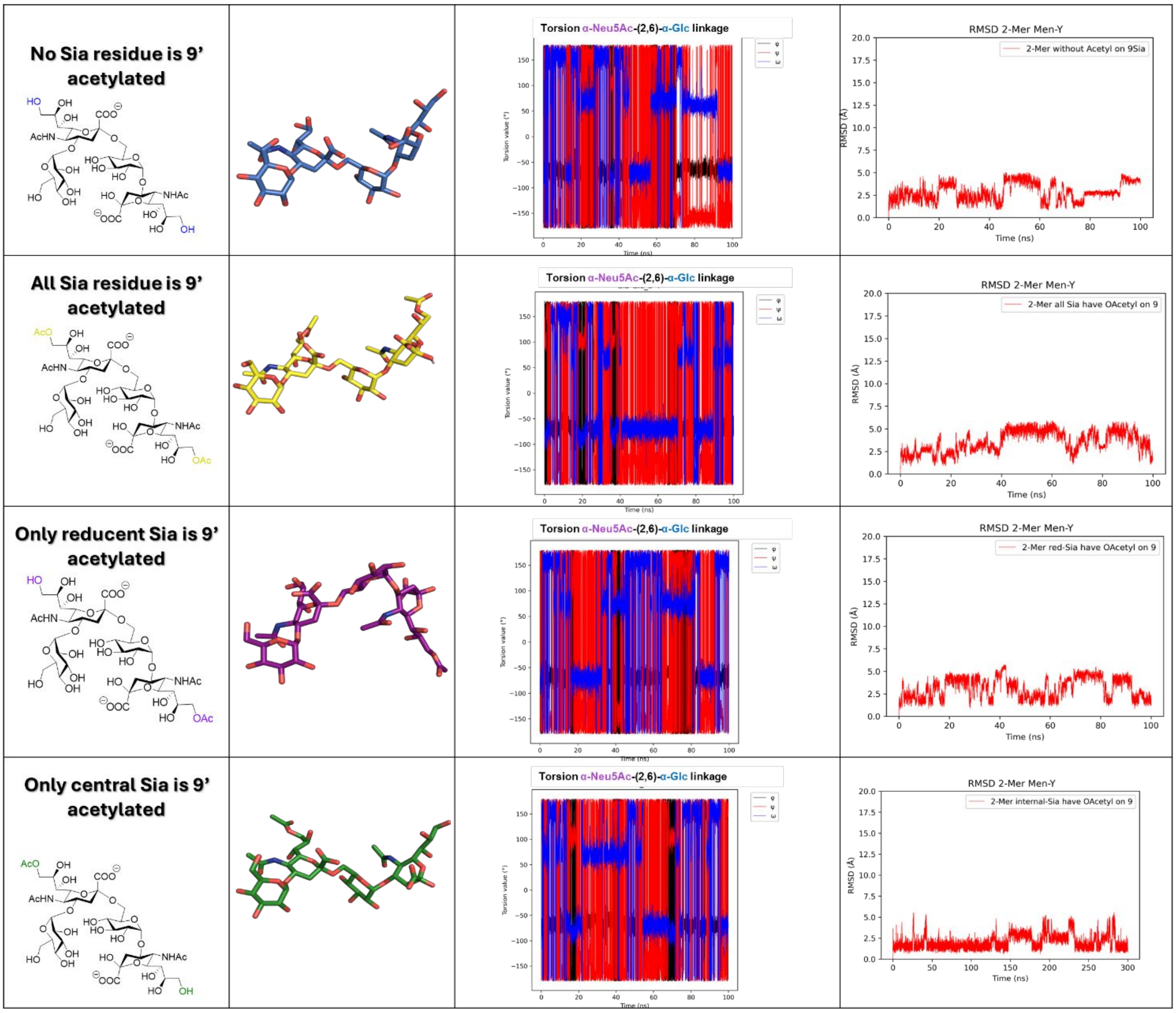
No Sia residue is 9' acetylated
All Sia residue is 9' acetylated
Only reducent Sia is 9' acetylated
Only central Sia is 9' acetylated
HO
OH
COO
AcHN
HO
NHAc
OOC
AcO
OAc
OH
Torsion α-Neu5Ac-(2,6)-α-Glc linkage
Torsion value (°)
Time (ns)
RMSD 2-Mer Men-Y
RMSD (Å)
2-Mer without Acetyl on 9Sia
2-Mer all Sia have OAcetyl on 9
2-Mer red-Sia have OAcetyl on 9
2-Mer internal-Sia have OAcetyl on 9

Figure S6.

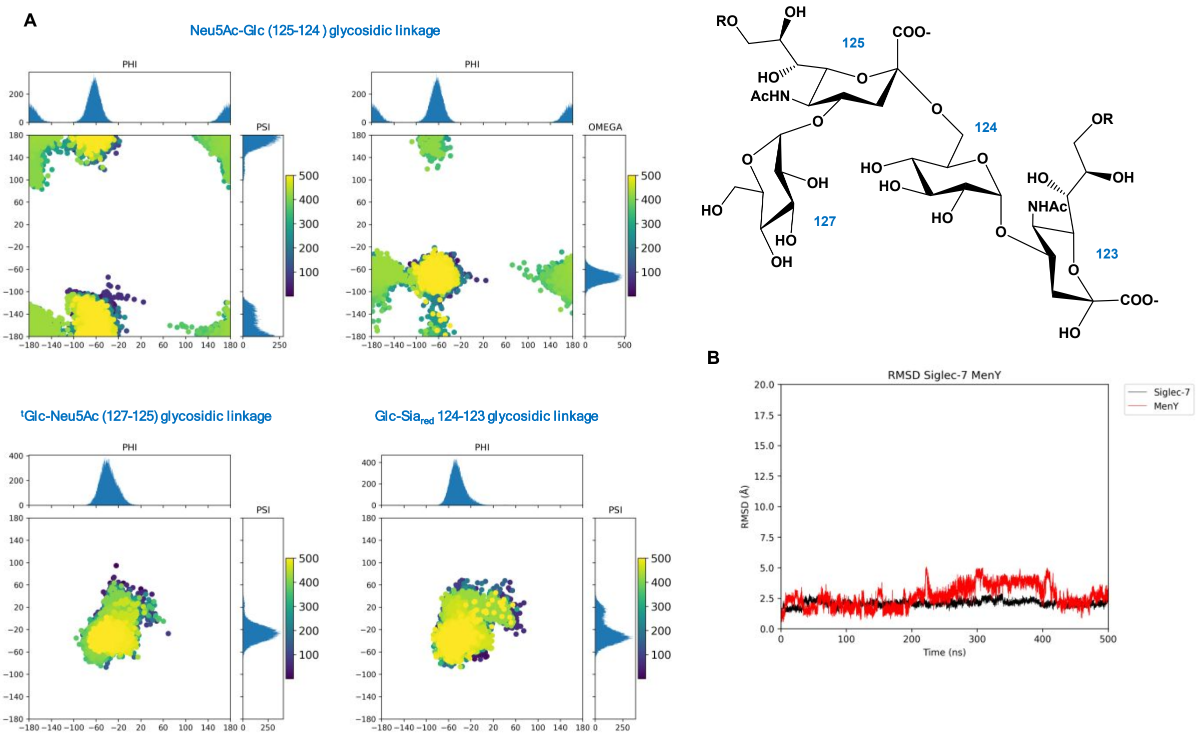

A
Neu5Ac-Glc (125-124) glycosidic linkage
PHI
PSI
OMEGA
ᵗGlc-Neu5Ac (127-125) glycosidic linkage
Glc-Sia$_{red}$ 124-123 glycosidic linkage
RO
OH
COO-
125
HO
AcHN
124
OR
127
123
NHAc
B
RMSD Siglec-7 MenY
Siglec-7
MenY
RMSD (Å)
Time (ns)

Figure S7

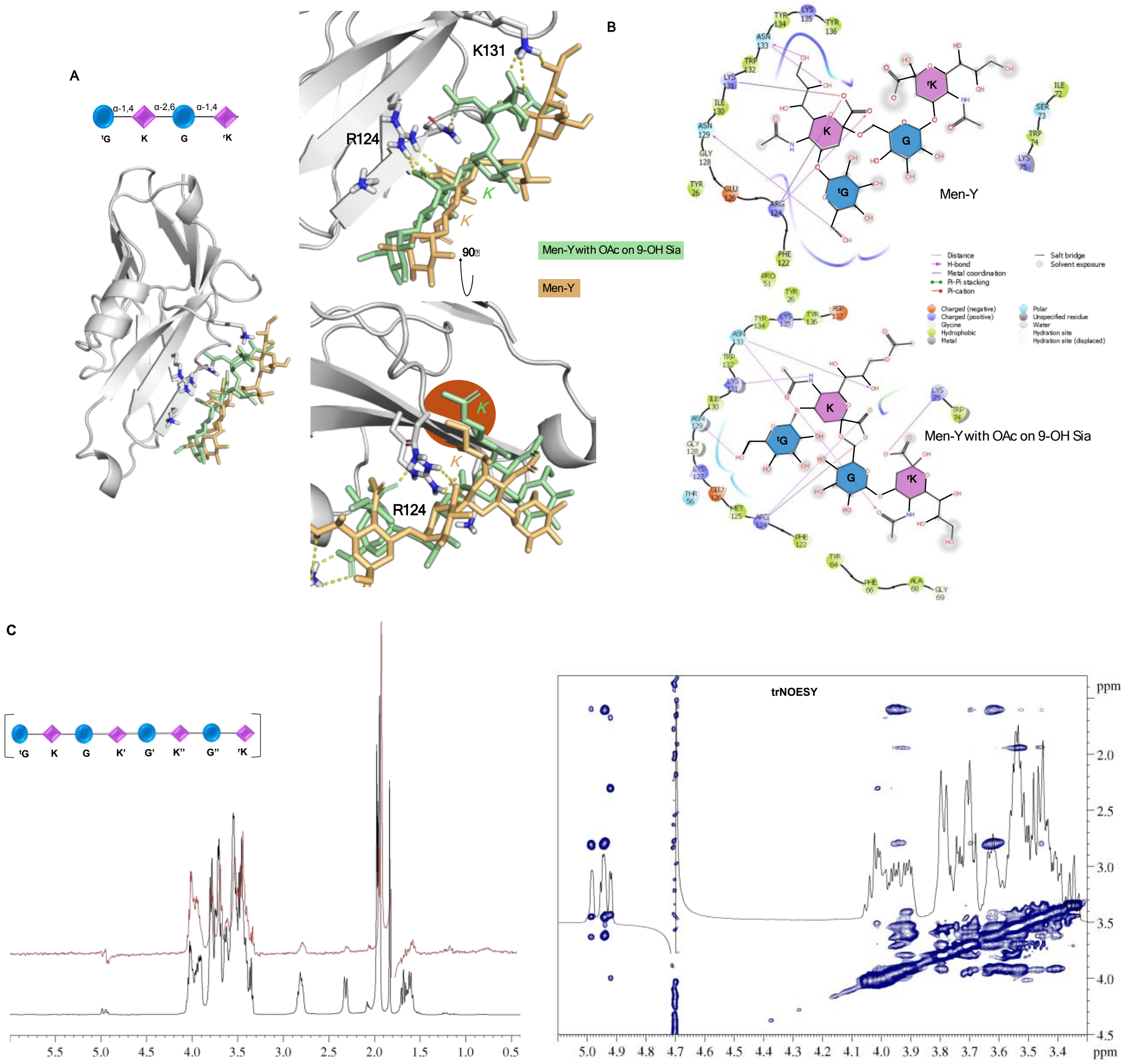

A
α-1,4
α-2,6
α-1,4
ᵗG
K
G
ʳK
B
K131
R124
K
K
90
Men-Y with OAc on 9-OH Sia
Men-Y
Men-Y
Men-Y with OAc on 9-OH Sia
C
ᵗG
K
G
K'
G'
K''
G''
ʳK
trNOESY
ppm

Figure S8

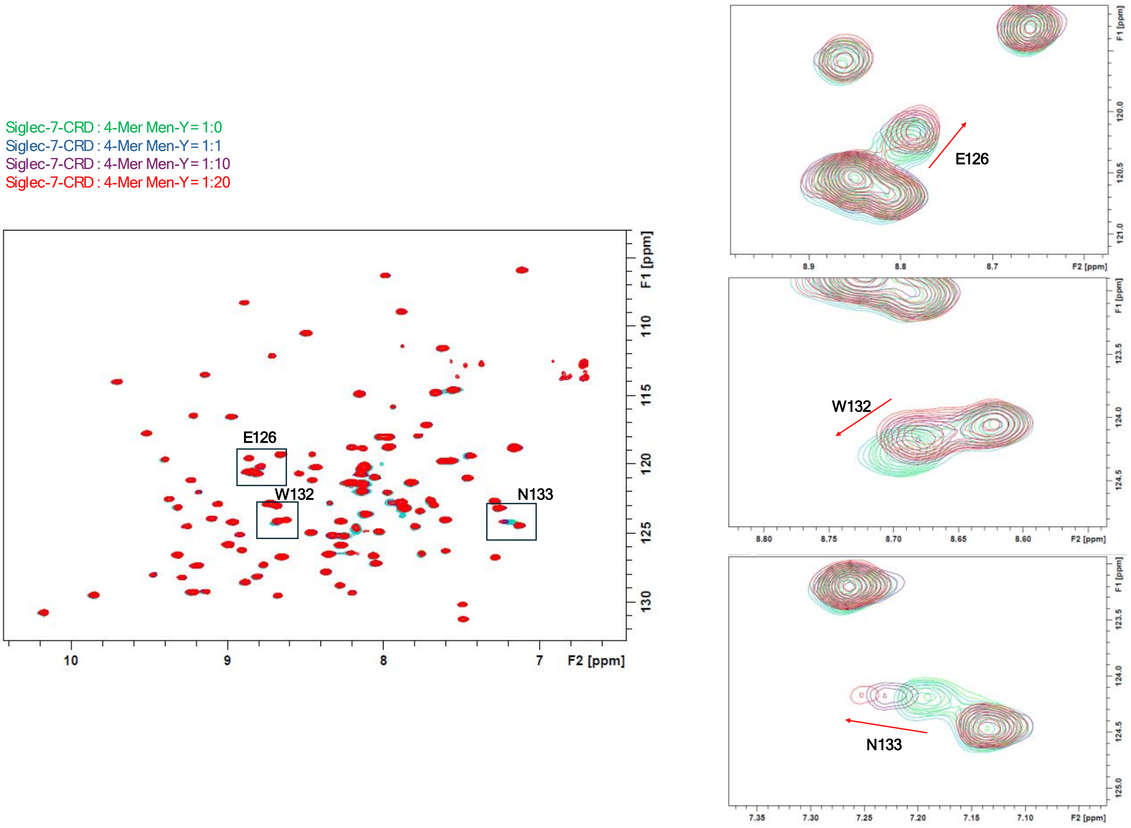
Siglec-7-CRD : 4-Mer Men-Y = 1:0
Siglec-7-CRD : 4-Mer Men-Y = 1:1
Siglec-7-CRD : 4-Mer Men-Y = 1:10
Siglec-7-CRD : 4-Mer Men-Y = 1:20
E126
W132
N133
F1 [ppm]
F2 [ppm]

Figure S9

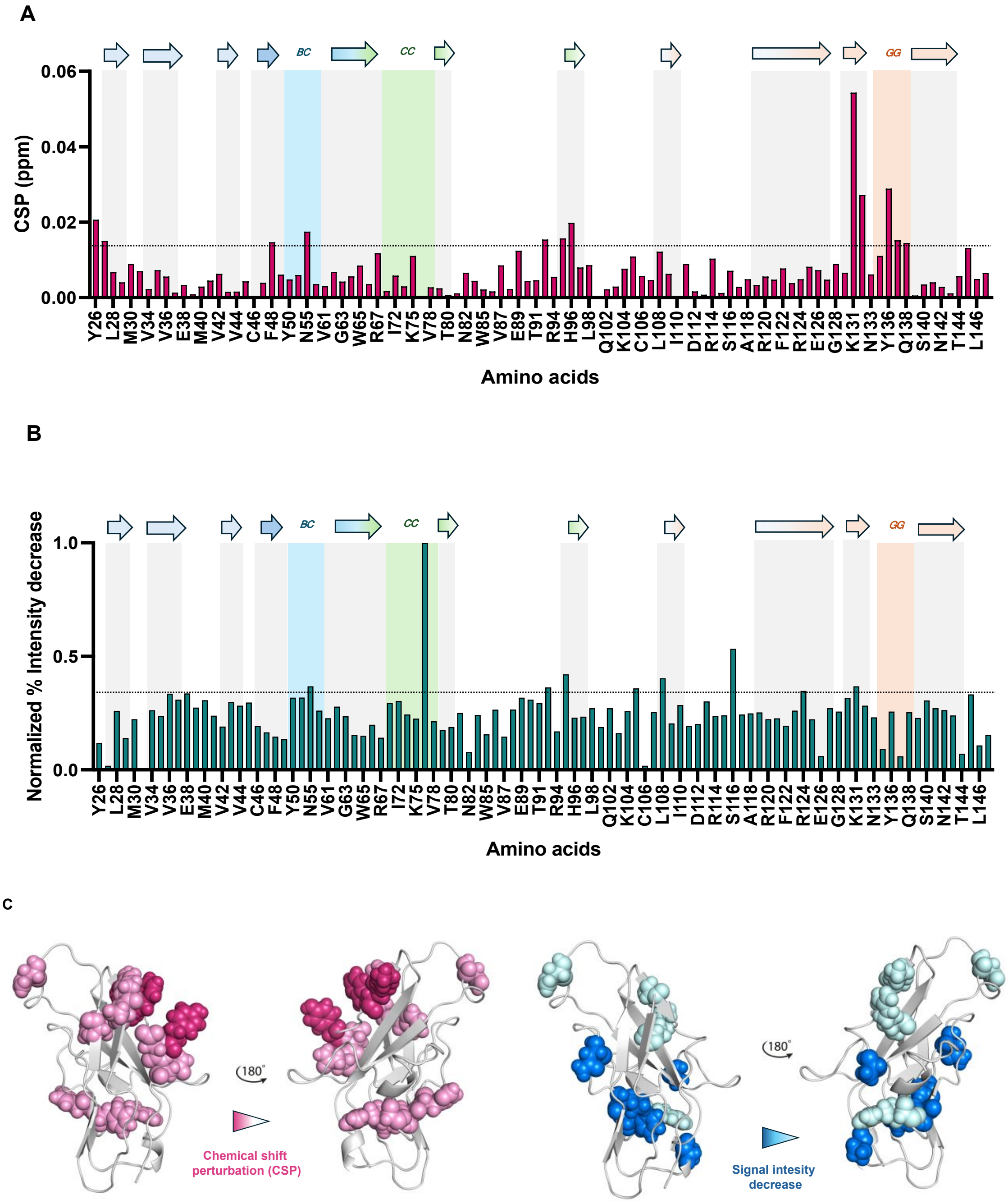
A
CSP (ppm)
Amino acids
B
Normalized % Intensity decrease
Amino acids
C
180°
Chemical shift perturbation (CSP)
Signal intesity decrease

Figure S10

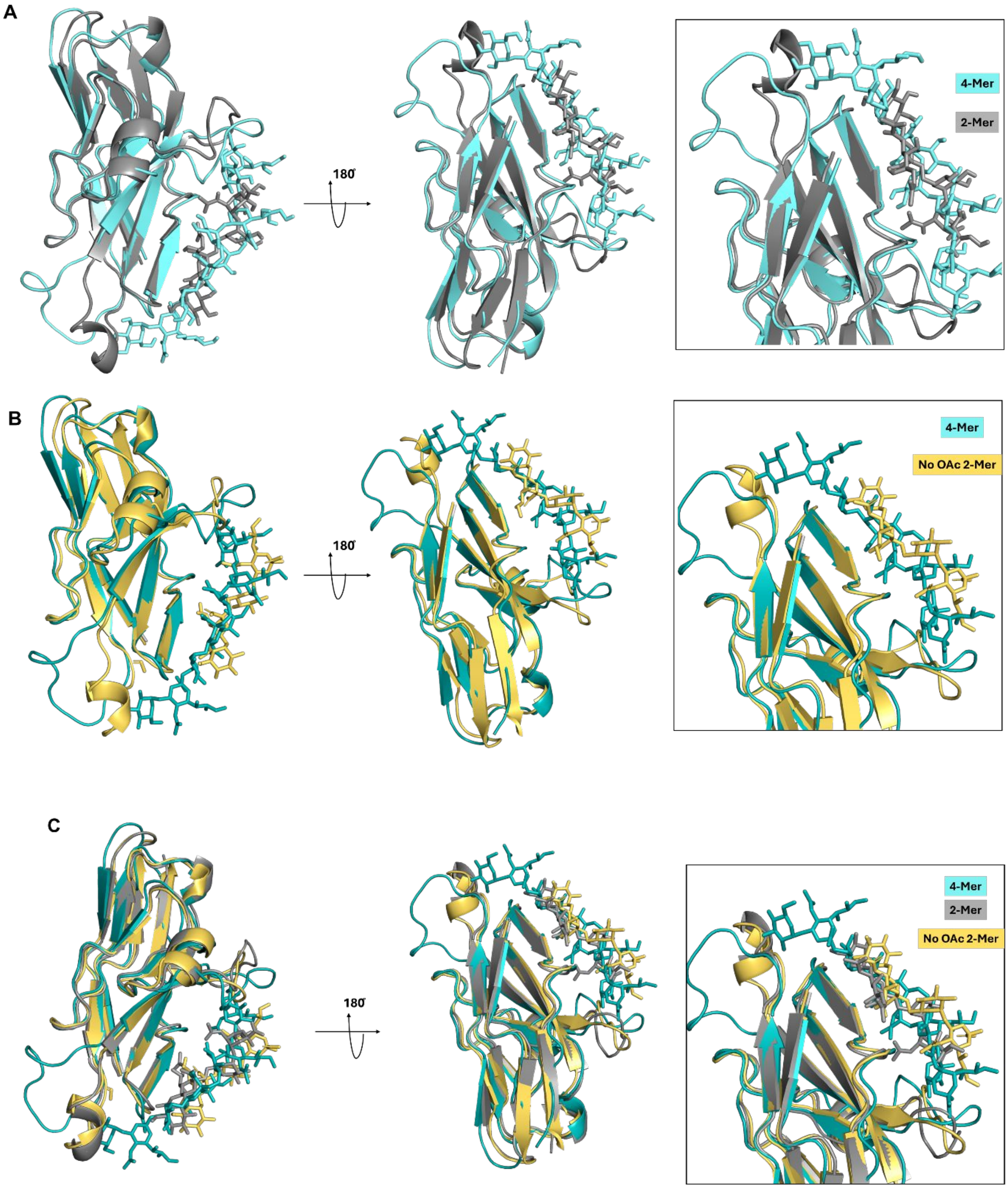
A
180
4-Mer
2-Mer
B
180
4-Mer
No OAc 2-Mer
C
180
4-Mer
2-Mer
No OAc 2-Mer

Figure S11

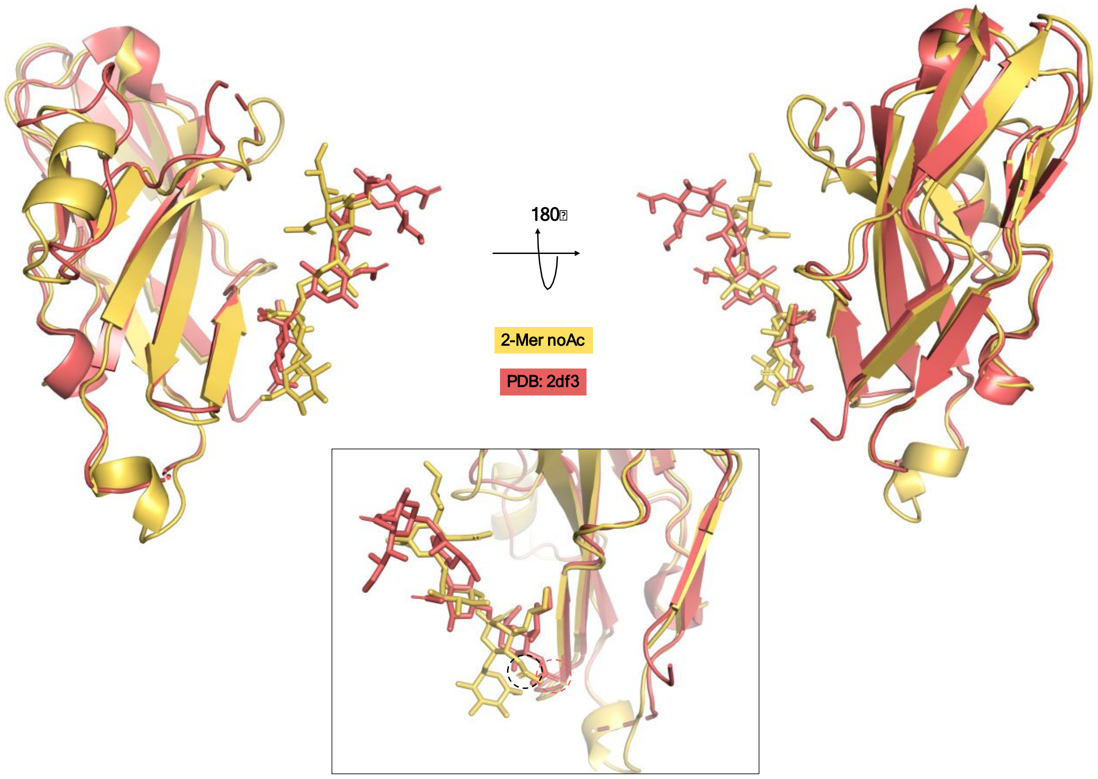

Supplement: Supplementary file 1 [file au5c00214_si_001.pdf]
